# Relationship between Hormonal Modulation and Gastroprotective Activity of Malvidin and Cyanidin Chloride: In Vivo and In Silico Approach

**DOI:** 10.3390/pharmaceutics14030565

**Published:** 2022-03-04

**Authors:** Melina Luzzi Zarricueta, Felipe Leonardo Fagundes, Quélita Cristina Pereira, Simone Queiroz Pantaleão, Raquel de Cássia dos Santos

**Affiliations:** 1 Laboratory of Pharmacology and Molecular Biology, Post Graduate Program in Health Sciences, Medical School, São Francisco University, Bragança Paulista, São Paulo 12916-900, Brazil; melina.luzzi@unesp.br (M.L.Z.); felipe.fagundes@unesp.br (F.L.F.); quelita.cristina@mail.usf.edu.br (Q.C.P.); 2 Laboratory of Computational Biology and Bioinformatics, Federal University of ABC, Av. dos Estados, 5001–Bangú, Santo André 09210-580, Brazil; simone.queiroz@ufabc.edu.br

**Keywords:** ethanol-induced gastric lesion, anthocyanidins, sex differences

## Abstract

Peptic ulcers are lesions that affect the gastrointestinal tract and that can be triggered by external factors such as alcohol use. This study investigated the gastroprotective role of two anthocyanidins, malvidin and cyanidin chloride, in an ethanol-induced gastric ulcer model in male and female mice (ovariectomized and supplemented with 17β-estradiol or not) and aimed to evaluate the effectiveness of anthocyanidins in preventing the formation of lesions and to identify the underlying mechanisms, while considering hormonal differences. Moreover, in silico comparative analysis was performed to predict the properties and biological behaviors of the molecules. We observed that the hormonal status did not interfere with the gastroprotective action of malvidin, although antioxidant mechanisms were modulated differently depending on sex. On the other hand, cyanidin showed gastroprotective activity at different doses, demonstrating that, for the same experimental model, there is a need to adjust the effective dose depending on sex. In silico analysis showed that, despite being structurally similar, the interaction with receptors and target proteins in this study (myeloperoxidase, superoxide dismutase, catalase, and reduced glutathione) differed between the two molecules, which explains the difference observed in in vivo treatments.

## 1. Introduction

Peptic ulcer (UP) is a gastrointestinal disease characterized by mucosal and submucosal erosions in the stomach and duodenum, affecting at least 10% of the world’s population each year [1]. Peptic ulcer is a multifactorial condition caused by factors such as *Helicobacter pylori* infection, use of non-steroidal anti-inflammatory drugs, stress, and chronic ethanol consumption [2]. Smoking and genetic background also contribute to the occurrence of UP and influence its progression and recurrence [3]. The factors that have protective effects against the development of UP in humans and animals include production of prostaglandins, adherent mucus, secretion of bicarbonate, high cell turnover in the gastrointestinal tract, adequate blood flow, and activity of antioxidant enzymes [4]. Other variables, such as sex, are implicated in the pattern of UP in men and women. Epidemiological studies indicate that women have 12% less chance of being affected by UP compared to men, but this difference is reduced with increase in age [5], which might be due to protective effects of the circulating hormones, particularly estradiol in females until menopause; thereafter, the production of hormones is considerably reduced. These sex differences are a new field of study in the areas of endocrinology and pharmacology [6]. Recent studies have indicated the differences in the biosynthesis of prostaglandins between males and females; for instance, female mice produced higher amounts of leukotriene B4 during the inflammation induced by Zymosan than that of male mice [7]. Another study has revealed that the healing of gastric ulcers in rats was dependent on sex, which showed different mechanisms of recovery after receiving a plant extract [8]. However, there are limited studies available based on sex differences in UP, mostly using hormonal supplementation as a pharmacological tool to normalize the hormonal status and to obtain more consistent results. The current pharmacological treatment for UP involves the use of antimicrobials when the disease is caused by *H. pylori* and anti-secretory drugs, including proton pump inhibitors (PPI) and H2 blockers [9], which reduce gastric acid secretion; however, anti-secretory drugs elevate the risk of hepatotoxicity, gastric adenocarcinoma, and infection by *Clostridium difficile* [10,11]. Considering these adverse effects, physicians and researchers seek drugs with gastroprotective action and with safe toxicological profile in both sexes. The phenolic compounds are a large group of bioactive molecules with pharmacological and nutraceutical properties used to treat various diseases, such as cancer, diabetes, cardiovascular and hepatic conditions [12,13]. Anthocyanidins are a subfamily of flavonoids that possess protective effects in obesity, inflammatory conditions, and neurodegenerative diseases [14,15]. These molecules can be found in natural sources, such as flowers, fruits, and seeds, and have in common a structure containing a flavylium B-ring and an absence of sugars, being the aglycone form of anthocyanins [16]. In this study, we aimed to investigate the gastroprotective action of two related anthocyanidins, malvidin and cyanidin chloride, in an ethanol-induced acute gastric lesion model in male and female mice (with or without hormonal supplementation with 17β-estradiol), and to examine the anti-inflammatory and antioxidant effects of these compounds; moreover, we used an in silico simulation approach to study the binding potential of the compounds, with some proteins studied in biochemical assays to better understand the mechanism underlying anthocyanidin treatment.

## 2. Materials and Methods

**Figure 1:**
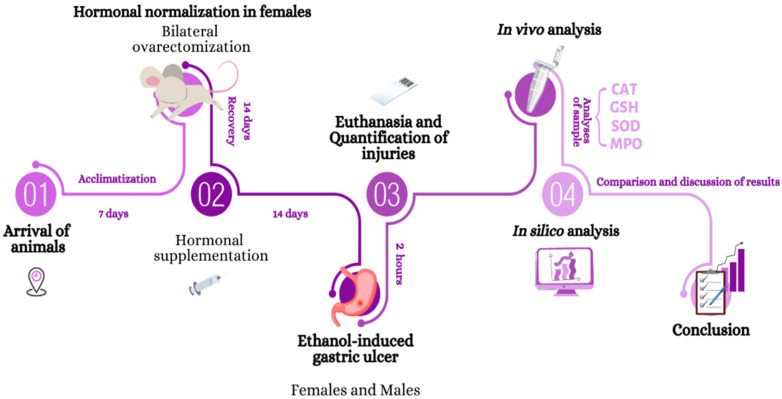
Schematic summary of the experimental design from the arrival of the animals to the analysis of the samples.

The following topics describe the techniques and methods used in this research which are illustrated in Figure 1, where the strategic view of how the work is structured is observed.

### 2.1. Chemicals

Malvidin and cyanidin hydrochloride (PubChem ID 69,512 and 68247, respectively) and 17β-estradiol (Pub Chem ID 5757) were purchased from Cayman Chemical Company (Ann Harbor, MI, USA). EDTA (PubChem ID 6049), catalase from bovine liver and protease inhibitor cocktail were taken from Thermo Fischer Scientific (Amherst, MA, USA). Purpald (PubChem ID 2723946), ortho-dianisidine dihydrochloride (PubChem ID 12309823), sodium hydroxide (PubChem ID 14798), potassium hydroxide (PubChem ID 14797), potassium dihydrogen phosphate (PubChem ID 516951), sodium fluoride (PubChem ID 5235), HTAB (PubChem ID 5974) and reduced glutathione (PubChem ID 124886) were obtained from Sigma Aldrich (Saint Louis, MO, USA). Lansoprazole (PubChem ID 3883) was obtained from Medley Sanofi (Campinas, SP, Brazil). Xylazine hydrochloride and ketamine (PubChem ID 68,554 and 1585, respectively) were obtained from Sespo Industry (Paulínia, SP, Brazil) and morphine from Cristália (Itapira, SP, Brazil). Hydrochloric acid (PubChem ID 313) was from J.T Backer (Xalostoc, Mexico). Absolute ethanol (PubChem ID 702), potassium periodate (PubChem ID 516896), and hydrogen peroxide (PubChem ID 784) were purchased from Synth Chemistry (São Paulo, SP, Brazil). Methanol (PubChem ID 887), Hypoxanthine (PubChem ID 135398638), nitrobluetetrazolium salt (PubChem ID 9281) and DTNB (PubChem ID 6254) were taken from Alfa Aesar (Tewksbury, MA, USA).

### 2.2. In Vivo Analysis

#### 2.2.1. Animals

Male and female Swiss mice were obtained from the Multidisciplinary Center for Biological Research in the Area of Laboratory Animal Science (CEMIB–Unicamp/SP), maintained in cages at a temperature of 22 ± 2 °C and 12 h light/dark cycle, and fed Presence diet; the animals had free access to water. When required, the animals were abstained from food for a maximum of eight hours. All experimental procedures were approved by and carried out in accordance with the Ethics Committee on the Use of Animals at São Francisco University (protocol number: 001.12.2017–male and 004.03.2020–female) and followed the Brazilian law of animal protection (Lei Arouca 11.794/2008) and the international recommendations for animal research (ARRIVE guidelines from NC3R).

#### 2.2.2. Groups and Doses Used

Animals were treated with malvidin chloride at doses of 2.5, 5, and 10 mg·kg^−1^, or cyanidin chloride at doses of 5, 10, and 20 mg·kg^−1^. Gastric ulcer was induced by absolute ethanol, and the animals were randomly divided into three groups: males, non-supplemented ovariectomized females, and ovariectomized females with hormonal supplementation with vehicle (0.9% saline), positive control (lansoprazole 30 mg·kg^−1^) [17] and test groups (malvidin and cyanidin). All treatments were administered orally. The fourth group consisted of untreated animals, white (males without any experimental procedure), and sham (ovariectomized females). After 60 min of administration of absolute ethanol, the animals were euthanized, and the samples of gastric tissue and blood were collected to assess macroscopic lesions and biochemical parameters.

#### 2.2.3. Bilateral Ovariectomy

Female mice (28-days old) underwent bilateral ovariectomy (OVX) [18] and received analgesia (morphine 3 mg·kg^−1^ [19]) and anesthesia (xylazine and ketamine (8 and 80 mg·kg^−1^, respectively). Subsequently, their dorsolateral sides were shaved and sterilized with iodine/ethanol (70%). For each ovary, a 0.5 cm incision was made through the skin and muscle; after localization, the ovaries were removed, and muscle and skin sutures were made using a nylon wire 4.0. Sham animals were handled similarly, and the ovaries were removed. After surgery, the mice were placed on heating pads for approximately 1 h for complete recovery. Other details, see Supplementary Material, Figures S1 and S2).

#### 2.2.4. Hormonal Supplementation

**Figure 2:**
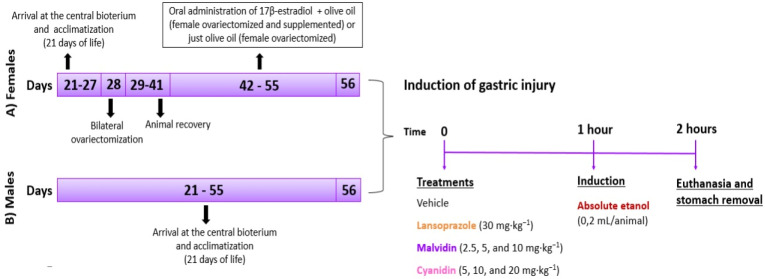
Schematic representation of the experimental protocol of the procedures performed in (**A**) female and (**B**) male mice.

Oral hormonal supplementation was provided for 14 days [20] using 17β-estradiol-3-benzoate (500 μg/kg) solubilized in olive oil [21]. Supplementation was initiated after complete recovery of ovariectomized animals (14 days) (Figure 2A).

#### 2.2.5. Gastric Ulcer Induced by Absolute Ethanol

After 14 days of supplementation with or without estradiol, the animals receiving pretreatment were divided into four groups (*n* = 5): vehicle, positive control, and test (malvidin or cyanidin). Eight hours before the test, the animals were deprived of food, but had access to water. The mice from the different experimental groups (male and female) received their respective treatments: vehicle (0.9% saline), lansoprazole (30 mg·kg^−1^), and test groups (malvidin at doses of 2.5, 5, and 10 mg·kg^−1^, or cyanidin at doses of 5, 10, and 20 mg·kg^−1^). The scheme of experimental procedures with males and females is summarized in Figure 2. Absolute ethanol at an oral dose of 0.2 mL (that possesses the ability to damage the gastric tissue, as described in the literature) was administered to the animal 60 min after pretreatment. After another 60 min, the animals were euthanized [22], and the gastric tissue and blood samples were collected for analysis of macroscopic lesions and assessment of oxidative and inflammatory parameters.

#### 2.2.6. Quantification of Ulcerative Lesions

The stomach was opened along the greater curvature and washed with saline solution (0.9% NaCl). The tissue was placed on glass plates and scanned using Epson digital scanner at a resolution of 1200 dpi. After scanning, the nonglandular portions of the stomach were removed and only the glandular tissue was used for the analysis of oxidative and inflammatory parameters. Ulcerative lesions were analyzed using the AvSoft Bio View Spectra 5 program with the following settings: 2% tolerance, selective scanning, disabled object count, and activated area measurement.

#### 2.2.7. Determination of Myeloperoxidase (MPO) Activity

Myeloperoxidase activity, an important marker of neutrophil infiltration, was determined using the Krawisz method [23]. Stomach strips previously stored at −80 °C were thawed and weighed using an analytical balance. The tissue (50 mg) was homogenized for 25 s in 1 mL of hexadecyltrimethylammonium bromide (HTAB) buffer using Polytron equipment at 4 °C. Afterwards, the samples were centrifuged for 10 min at 10.624 rcf at 4 °C. The sample supernatant was then collected to quantify the enzymatic activity by measuring the absorbance at 450 nm using Multiskan microplate reader.

#### 2.2.8. Determination of Reduced Glutathione (GSH) Level

Glutathione determination was based on the Faure protocol [24], where stomach samples stored at −80 °C were solubilized in extraction buffer at a ratio of 1:4 and 1% of protease inhibitor cocktail. Then, the material was homogenized for 25 s using Polytron equipment at 4 °C. The samples were centrifuged for 45 min at 20.817 rcf at 4 °C and diluted 1:10 with phosphate-buffered saline (PBS). The level of GSH present in the tissue was determined using a standard curve of reduced l-glutathione and by measuring the absorbance at 414 nm using a microplate reader.

#### 2.2.9. Determination of Superoxide Dismutase (SOD) Activity

Stomach samples stored at −80 °C were weighed and homogenized in extraction buffer at a ratio of 1:4 and 1% of protease inhibiting cocktail. Then, the samples were processed for 25 s using Polytron equipment and centrifuged for 45 min at 20.817 rcf at 4 °C; subsequently, the supernatant was collected and diluted 1:20 using (PBS). A solution containing hypoxanthine, xanthine oxidase, and nitro blue tetrazolium salt at the ratio of 1:1:1 was added to a microplate containing samples, which was then inserted into the Glomax 96-well plate reader, which performed a kinetic reading for 10 min at a wavelength of 560 nm [25].

#### 2.2.10. Determination of Catalase (CAT) Activity

Stomach samples stored at −80 °C were solubilized in extraction buffer at a ratio of 1:4 and 1% of protease inhibitor cocktail. The samples were then processed for 25 s using Polytron equipment and centrifuged for 45 min at 20.817 rcf at 4 °C to form a pellet; the sample supernatant was collected. To determine the CAT activity, 20 µL of sample previously diluted with (PBS) 1:10 was added to a plate containing 100 µL of assay buffer and 30 µL of methanol. Next, a standard curve was prepared with known concentrations of formaldehyde (0–75 µM) and a stock solution of catalase from bovine liver (10 mg/mL) in two control wells. Subsequently, 20 µL of H_2_O_2_ (35.3 mM) was added to the plate and incubated at room temperature (22 °C) for 20 min. After this period, 20 µL of 10 M KOH and 45 µL of 34.2 mM Purpald reagent (4-amino-3-hydrazino-5-mercapto-1,2,4-triazole) were added to the wells, followed by incubation at room temperature (22 °C) for 10 min. Finally, 15 µL of KIO_4_ (65.2 mM) was added, and the plate was placed on a shaker for 5 min. The absorbance was determined using a Multiskan microplate reader at a wavelength of 540 nm [26].

### 2.3. In Silico Analysis

#### 2.3.1. Simulation of the Pharmacokinetic Profile Using the SwissADME Server

The pharmacokinetic profiles of the anthocyanidins cyanidin and malvidin were drawn using the SwissADME [27,28,29] server (available online: http://www.swissadme.ch/index.php last accessed on 22 September 2021), considering the structural data of the molecules in SMILES (simplified molecular input line entry specification) format, which were used to obtain the physicochemical descriptors related to oral bioavailability (lipophilicity, molecule size, polarity, solubility, flexibilization, and unsaturation of the molecule) and the prediction of permeation of the blood-brain barrier (BBB) and the gastrointestinal tract (HIA) represented by the BOILED-Egg plot. Moreover, complementary aspects, such as molecular weight (MW), molecular refractivity (MR), polar surface area (PSA), polar topological surface area (TPSA), lipophilicity (partition coefficient between n-octanol and water (log Po/w)), and water solubility, were determined to verify whether the anthocyanidins qualifies the drug-likeness filter, indicating structural similarity to marketed drugs. In addition, the following filters implemented in the tool were used: (a) Lipinski, (b) Ghose, (c) Veber, and (d) Muegge, as well as the numerical indication of synthetic accessibility (ranging from 1: very easy to 10: very difficult), and probing for structural alerts known as (a) pan-assay interference compounds (PAINS) that are indicative of promiscuous compounds and (b) BRENK that identifies fragments supposed to be toxic, chemically reactive, metabolically unstable, or to carry properties responsible for poor pharmacokinetics.

#### 2.3.2. Simulation of Pharmacokinetic Property Prediction Using the pkCSM Server

The subsequent step involved submitting the SMILES of the anthocyanidins to the pkCSM [30] server (available online: http://biosig.unimelb.edu.au/pkcsm/ last accessed on 26 October 2021) for analysis of their ADMET profiles based on the graphical signature of the molecule, providing data on (a) absorption: water solubility, Caco2 permeability, human intestinal absorption, skin permeability, P-glycoprotein substrate, and P-glycoprotein inhibitor; (b) distribution: human steady-state volume of distribution (VDss), unbound (human) fraction, blood-brain barrier permeability, central nervous system (CNS) permeability; (c) metabolism: as CYP2D6/CYP3A4 substrate, inhibition of CYP1A2/CYP2C19/CYP2C9/CYP2D6/CYP3A4; (d) excretion: total and by renal substrate OCT2); (f) toxicity: AMES toxicity, maximum tolerated dose (human), inhibition of hERG and hERG II, acute oral toxicity in rats (LD50), chronic oral toxicity in rats (LOAEL), hepatotoxicity, skin sensitization, toxicity to *Tetrahymena pyriformis* and minnow).

#### 2.3.3. Simulation of Toxic Substructure Using eMolTox Server

Although we obtained toxicity prediction data using the pkCSM4 server, we incorporated a joint toxic substructure (toxicophores) analysis in this study using the eMolTox [31] server (available online: http://xundrug.cn/moltox last accessed on 26 October 2021) to check for possible specific structural alerts such as hepatotoxicity, cardiotoxicity, mutagenicity, genotoxicity, carcinogenicity, cytotoxicity, structural toxic alerts, mitochondrial toxicity, nephrotoxicity, CNS toxicity, respiratory toxicity, reproductive toxicity, skin sensitization, cytochrome P450 interaction, and acute oral toxicity, to determine the safety profile regarding these aspects; the input was the SMILES of each molecule, as mentioned previously.

#### 2.3.4. Simulation of the Probable Biological Targets Using the SwissTargetPrediction Server

Based on the structures of the anthocyanidins cyanidin and malvidin, a prediction was made using the computational tool SwissTargetPrediction [32] (available online: http://www.swisstargetprediction.ch/ last accessed on 25 October 2021) of the possible biological targets that present some degree of binding affinity with such molecules. This prediction was based on the combination of 2D and 3D similarity with a library of 370,000 known actives, including approximately more than 3000 proteins; the data obtained can guide further analysis such as molecular docking focusing on receptor-ligand molecular interactions. The data obtained consisted of a list of possible biological targets, followed by their Uniprot ID [33] and ChEMBL ID [34,35,36] codes (available online: https://www.uniprot.org/ and https://www.ebi.ac.uk/chembl/, respectively last accessed on 26 October 2021), which can be accessed for detailed information on the target. The list presented is ranked according to the probability percentage of matching with the proteins; the data are graphically represented.

#### 2.3.5. Simulation of Molecular Docking Using the Achilles Blind Docking Server

To date, the mechanisms of action of anthocyanidins (cyanidin and malvidin) have not been described when coupled to the biological targets of proteins such as SOD, CAT, GSH, and MPO. Since it has not been reported in the literature which regions (binding sites) and which amino acid residues would be important to establish molecular interactions in the coupling of these anthocyanidins to these proteins, there was a need to perform the blind docking technique to first track which regions would be the most favorable for binding (according to the criterion of binding energy values), employing the method called Vina_vision, which uses a pose clustering algorithm based on the traditional AutoDock Vina software [32,33] implemented in Achilles Blind Docking Server [34] (available online: http://bio-hpc.eu/software/blind-docking-server/ last accessed on 2 November 2021), which first calculates the fit of the molecules across the protein surface to find the points with the best binding affinities, then proceeds with the clustering of the poses, where the best affinity is considered as the representation of the cluster. The crystallographic structures used in this study were: SOD with PDB code 1N0J [35]; CAT with PDB code 1F4J [36], GSH with PDB code 3SQP [37] and MPO with PDB code 7LAE [38], all made available in the Protein Database PDB [39,40] (available online: https://www.rcsb.org/ last accessed on 2 November 2021). The protein and ligand input files were generated in pdbqt format, with AutoDock Vina parameterization. The generated conformations were evaluated under the following criteria: (a) conformation/geometry; (b) frequency of binding energies of individual poses and groups; (c) distances between clusters in angstrom; (d) contribution to the overall binding affinity of the pose, considering the terms: Gauss1*weight_gauss1, Gauss2*weight_gauss2, Repulsion*weight_repulsion, Hydrophobic*weight_hydrophobic, Hydrogen*weight_hydrogen, TORSDOF*weight_rot; (e) detailing the different contributions of each atom in the ligand to the overall binding affinity of the pose; (f) ligand/receptor molecular interactions.

### 2.4. Statistical Analysis

The results are expressed as the mean ± SEM. The Kolmogorov-Smirnov test was used to verify data normality in all data sets. Statistical evaluation of the results was carried out using parametric analysis of one-way of variance (ANOVA) to verify the difference between the means followed by the Dunnett’s test. Statistical analyses were performed using GraphPad Prism 8.0 (San Diego, CA, USA), and a *p*-value less than 0.05 was considered significant.

## 3. Results

### 3.1. In Vivo

#### 3.1.1. Absolute Ethanol-Induced Gastric Lesions

**Figure 3:**
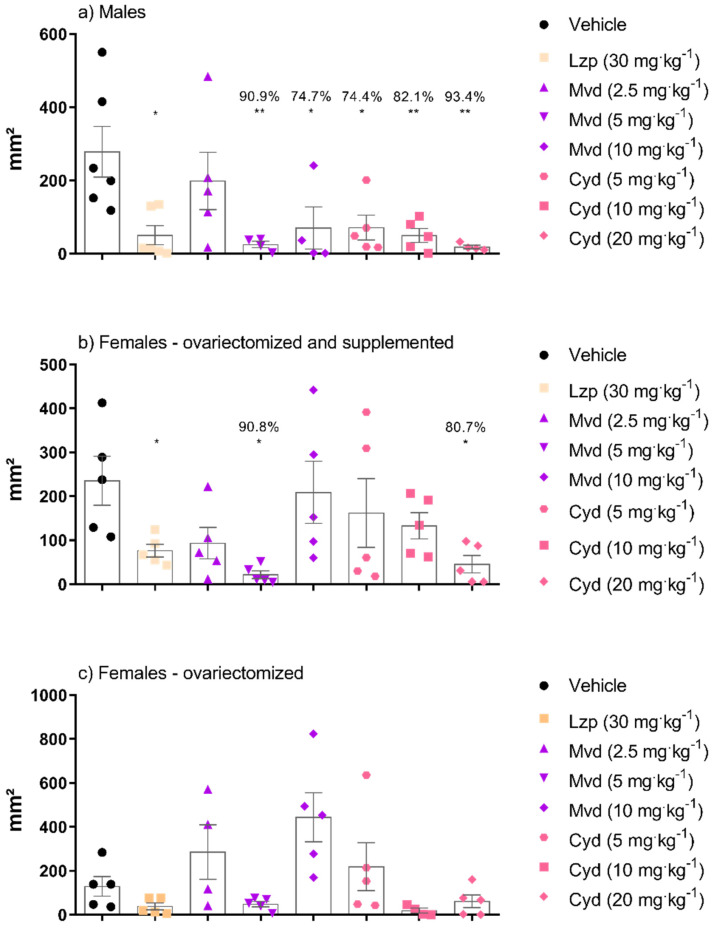
Effect of oral treatment with malvidin or cyanidin chloride in the prevention of gastric lesions induced by ethanol. The results indicate the mean lesion area for each treatment, in the different groups: (**a**) male animals, (**b**) female animals ovariectomized and supplemented with 17β -estradiol (500 μg/kg) and (**c**) group of ovariectomized female animals without supplementation. Data are presented as mean ± S.E.M. ANOVA followed by the Dunnett’s test; where * *p* < 0.05 and ** *p* < 0.01, compared to the respective vehicle group. Percentage numbers indicate inhibition of lesion formation.

**Figure 4:**
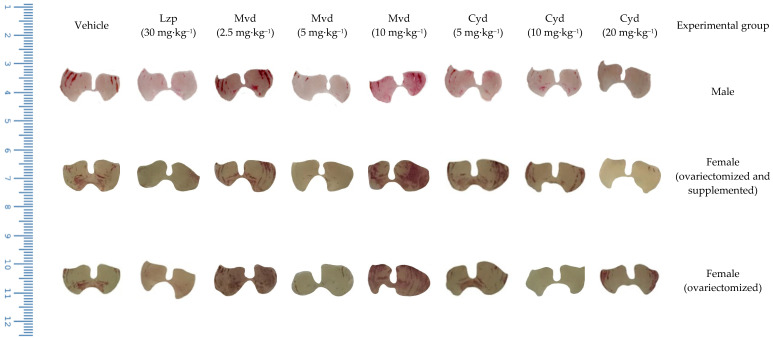
Representative panel with images of mice stomachs treated with vehicle, lansoprazole, malvidin, and cyanidin and submitted to the protocol of gastric ulcer induced by ethanol.

An experimental model of ethanol-induced gastric ulcer was used to investigate the gastroprotective effect of malvidin and cyanidin. Figure 3 shows the results of macroscopic analysis, which suggest that malvidin was effective in inhibiting the formation of lesions in all hormonal scenarios, showing significant gastroprotection at the same dose of 5 mg·kg^−1^ in male and female mice. On the other hand, cyanidin showed macroscopic gastroprotection in males (all doses were effective) and supplemented females, but the dose needed for gastroprotection differed according to sex (5 and 20 mg·kg^−1^, respectively). Figure 4 shows a representative panel of rats treated according to the protocol followed in this work.

Details of the effects of oral treatment with malvidin or cyanidin chloride on the prevention of ethanol-induced gastric lesions (results presented as: mean ± standard error of the mean) are shown in Table S1 (Supplementary Material).

#### 3.1.2. Malvidin and Cyanidin Showed Anti-Inflammatory Activity in Stomach Samples of Ethanol-Treated Mice

**Figure 5:**
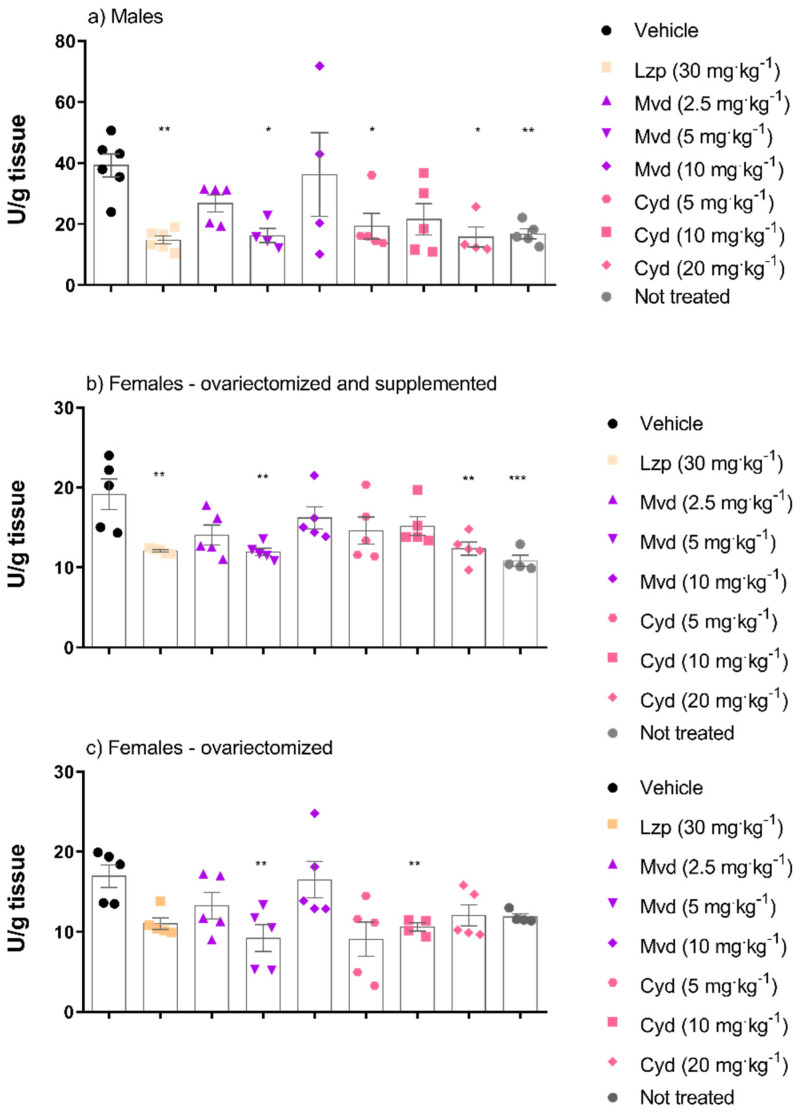
Effects of the malvidin and cyanidin chloride treatment on the MPO activity in the gastric lesions induced by ethanol in the different groups: (**a**) male animals, (**b**) female animals ovariectomized and supplement with 17β-estradiol (500 μg/kg) and (**c**) group of ovariectomized female animals without supplementation. Data are presented as mean ± S.E.M. ANOVA followed by the Dunnett’s test; where * *p* < 0.05, ** *p* < 0.01 and *** *p* < 0.001, compared to the respective vehicle group.

To investigate the mechanisms involved in the gastroprotective effect of anthocyanidins, MPO activity was analyzed. Figure 5 shows that treatment with malvidin was effective in inhibiting MPO activity significantly when administered orally at a dose of 5 mg·kg^−1^. Treatment with cyanidin was effective in inhibiting MPO in all groups, but at different doses: 5 and 20 mg·kg^−1^ in the male mice group, 20 mg·kg^−1^ in ovariectomized and supplemented female mice group, and 10 mg·kg^−1^ in ovariectomized group.

#### 3.1.3. Anthocyanidins Showed Antioxidant Activity in Stomach Samples of Ethanol-Treated Mice

##### SOD Activity Analysis

**Figure 6:**
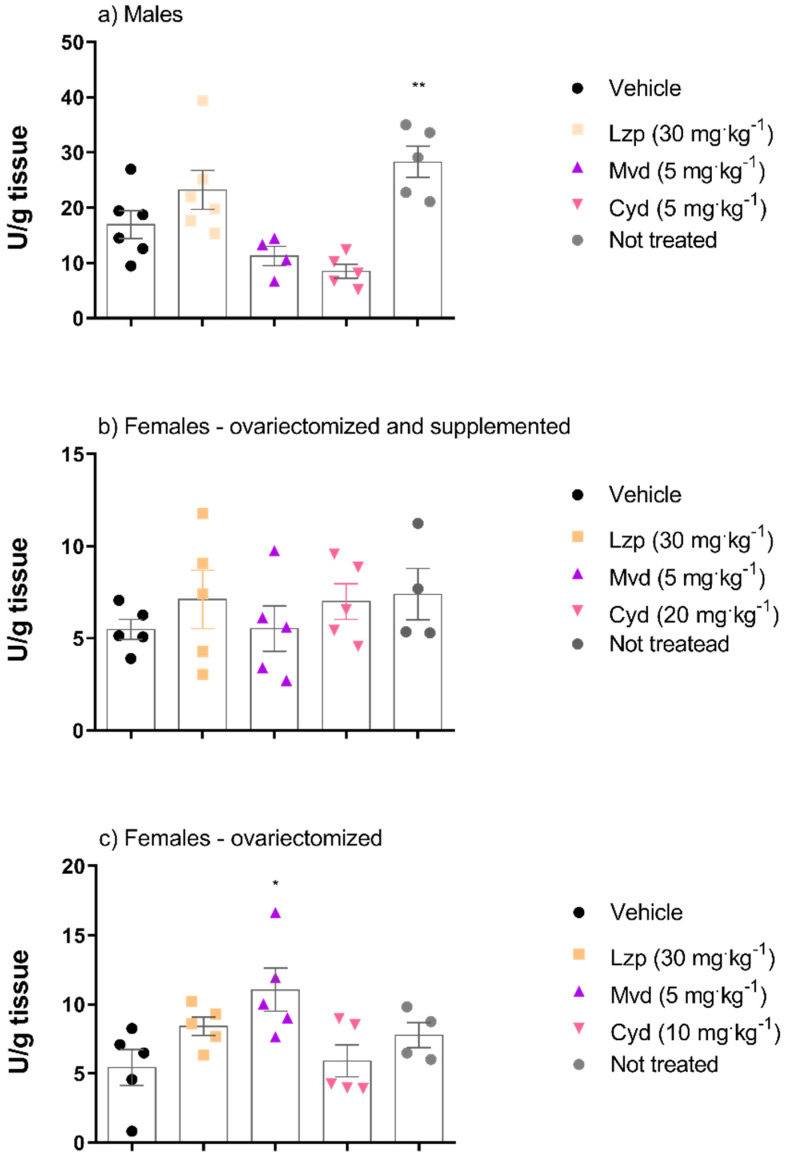
Effects of the malvidin or cyanidin chloride treatment on the SOD activity in the gastric lesions induced by ethanol in the different groups: (**a**) male animals, (**b**) female animals ovariectomized and supplemented with 17β-estradiol (500 μg/kg) and (**c**) group of ovariectomized female animals without supplementation. Data are presented as mean ± S.E.M. ANOVA followed by the Dunnett’s test; where * *p* < 0.05 and ** *p* < 0.01, compared to the respective vehicle group.

To verify whether anthocyanidins exert antioxidant effects, we performed SOD analysis. Figure 6 shows the results of the SOD activity analysis. Malvidin modulated this activity in ovariectomized female mice group, but not in ovariectomized and supplemented animals. No significant changes in SOD activity were observed in both male and female mice treated with cyanidin. 

##### CAT Activity Analysis

**Figure 7:**
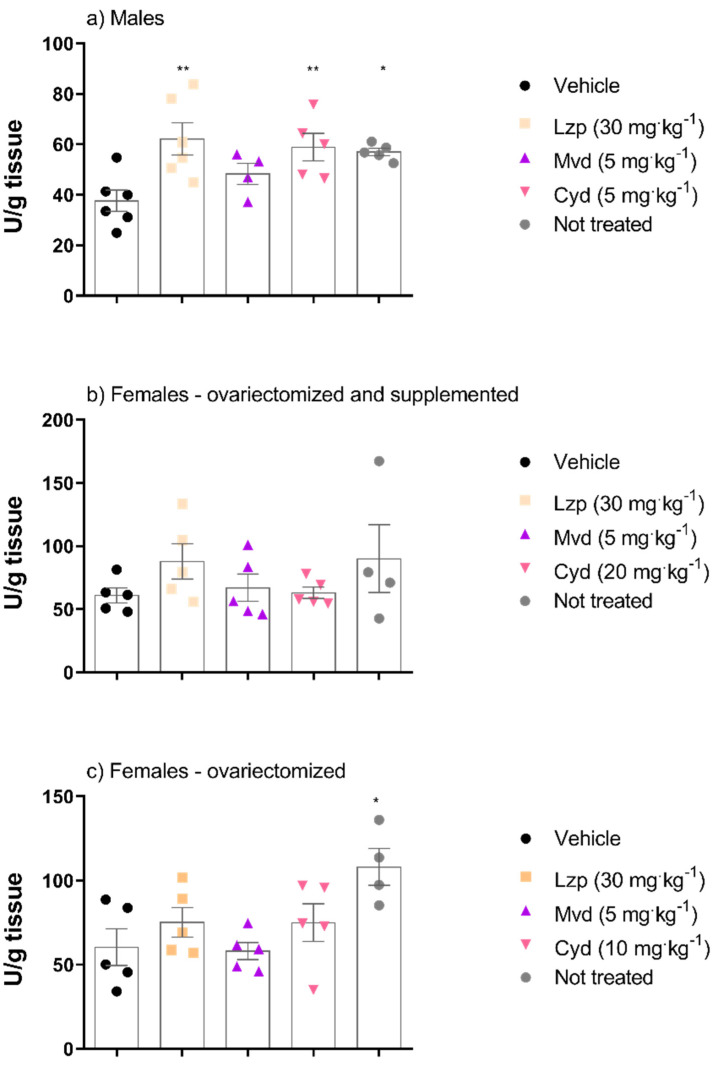
CAT activity in the gastric tissue of mice treated with malvidin and cyanidin chloride before gastric lesions induced by absolute ethanol in the different groups: (**a**) male animals, (**b**) female animals ovariectomized and supplemented with 17β-estradiol (500 μg/kg) and (**c**) group of ovariectomized female animals without supplementation. Data are presented as mean ± S.E.M. ANOVA followed by the Dunnett’s test; where * *p* < 0.05 and ** *p* < 0.01, compared to the respective vehicle group.

Treatment with cyanidin (5 mg·kg^−1^) in male mice, but not in female mice, was responsible for the increase in CAT activity in this experimental model. Malvidin treatment did not show alteration in CAT activity in any experimental group (Figure 7).

##### Analysis of GSH Level

**Figure 8:**
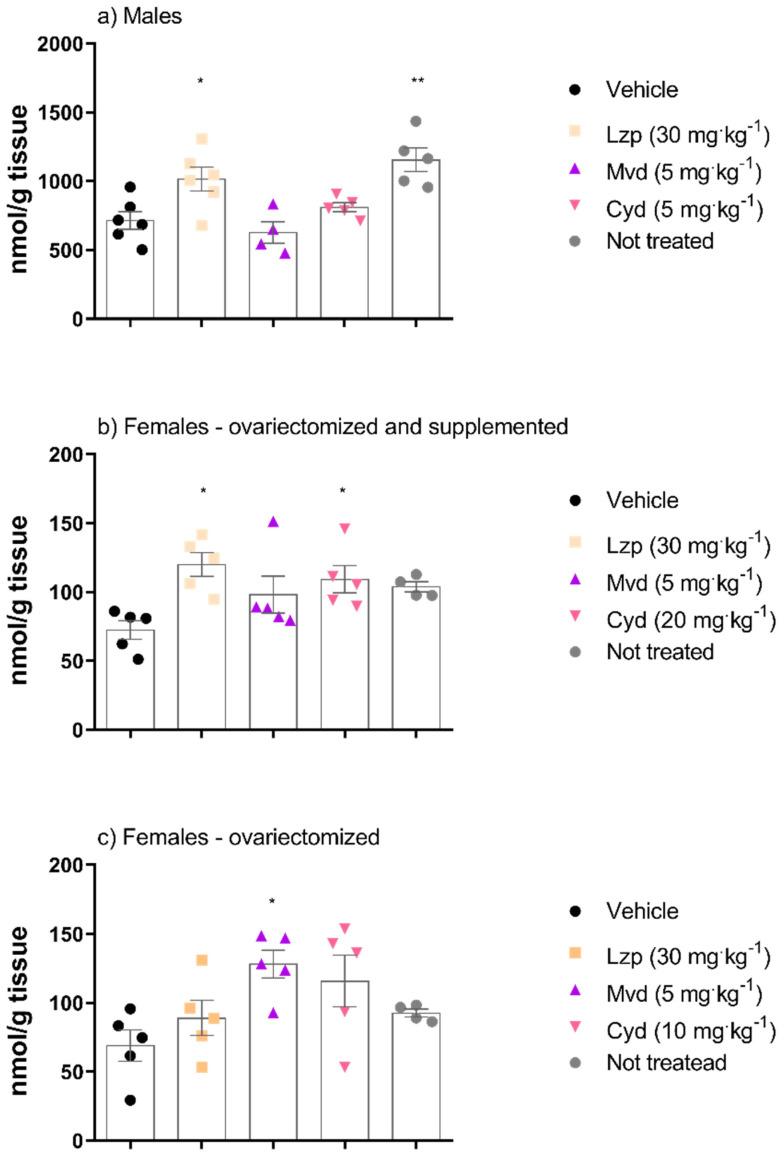
The GSH levels in the stomach of mice treated with malvidin and cyanidin chloride before gastric lesions induced by absolute ethanol in the different groups: (**a**) male animals, (**b**) female animals ovariectomized and supplemented with 17β-estradiol (500 μg/kg) and (**c**) group of ovariectomized female animals without supplementation. Data are presented as mean ± S.E.M. ANOVA followed by the Dunnett’s test; where * *p* < 0.05 and ** *p* < 0.01, compared to the respective vehicle group.

Oral treatment with malvidin at a dose of 5 mg·kg^−1^ significantly increased the level of GSH in the stomach of ovariectomized female mice (Figure 8). Moreover, an increase in the level of GSH was observed only in the group of ovariectomized and supplemented animals treated with cyanidin at a dose of 20 mg·kg^−1^. However, treatment with either of the molecules did not alter GSH level in male mice.

### 3.2. In Silico

#### 3.2.1. Pharmacokinetic Profile of Malvidin and Cyanidin Chloride

**Figure 9:**
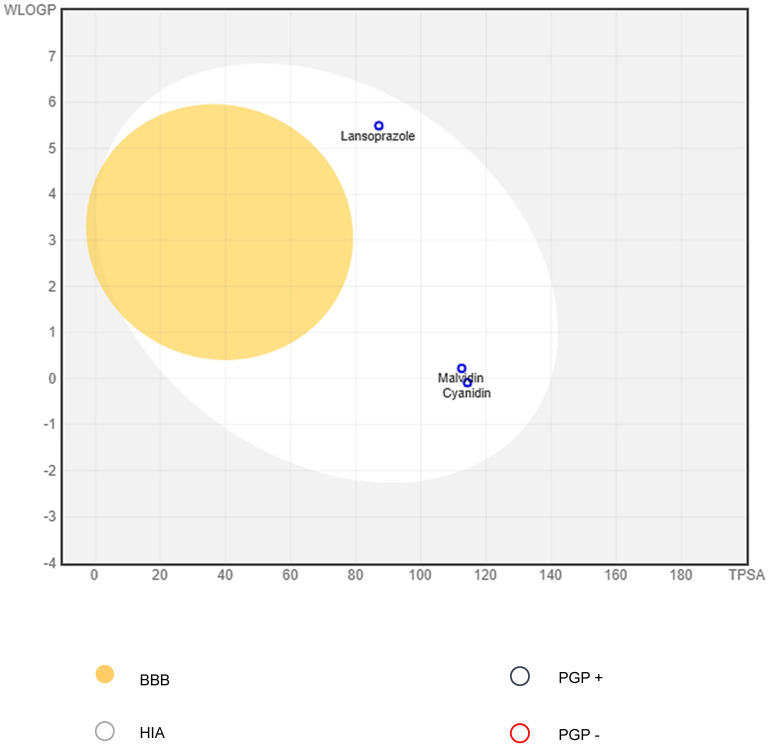
Comparative graphical representation (BOILED-Egg) for the molecules: lansoprazole, malvidin chloride and cyanidin chloride.

The reference molecule lansoprazole was used as a positive standard for analysis of ADMET profiles of anthocyanidins in silico using the SwissADME [27,28,29] server. According to the BOILED-Egg representation, Figure 9 shows the safe limits of human intestinal absorption permeability (HIA) (located in the white region) and BBB permeability (located in the yellow region); the three molecules showed compatible results of high HIA and no BBB permeability, indicating safety because the anthocyanidins actions are exclusively related to the gastrointestinal process. This graph correlates the data regarding the acceptable limits for LogP (between −2.3 and +6.8) and TPSA (less than 142 Å^2^); lansoprazole showed TPSA of 87.08 Å^2^ and LogP of 5.5, whereas malvidin showed LogP of 0.08, and cyanidin had TPSA of 114.29 Å^2^ and LogP of −0.45; the values indicate high gastrointestinal absorption of the anthocyanidins.

**Figure 10:**
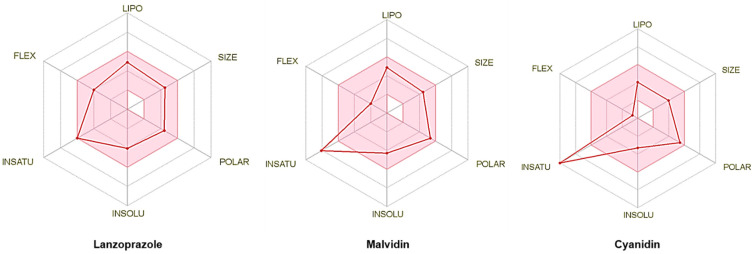
Oral bioavailability radar for the molecules lansoprazole, malvidin, and cyanidin. The colored area of the graph represents physicochemical space for optimum oral bioavailability. LIPO (lipophilicity): −0.7 < XLOGP3 < +5.0; SIZE: 150 g/mol < MV < 500 g/mol; POLAR (polarity): 20 Å^2^ < TPSA < 130 Å^2^; INSOLU (insolubility): 0 < LOG S (ESOL) < 6; INSATU (establishment): 0.25 < CSP3 fraction < 1; FLEX (flexibility): 0 < number of rotatable connections < 9.

Figure 10 shows the oral bioavailability radars; despite being structurally similar, malvidin and cyanidin have physicochemical characteristics with significant point differences, which may contribute to the diverse ADME profile. In addition to the bioavailability radar, the SMILES of these molecules were submitted to the pkCSM [28] server to check their profiles in detail; Tables S2 and S3 (Supplementary Material) contains all data of predictive analysis.

To evaluate toxicity of the possible fragments of the molecules in the body, the SMILES of the three molecules of interest were submitted to the eMolTox [31] server. The fragmentation of the molecules was simulated by confronting toxic structural alerts from the toxic fragment library of the eMolTox [31] server computational tool for drugs that have presented these problems. A prominent parameter of this prediction, the verification of toxicity or mutagenic effects by the AMES test, indicated that niether anthocyanidins showed any mutagenic factor; surprisingly, mutagenic toxicity has been detected for lansoprazole, a drug used for the treatment of peptic ulcer.

#### 3.2.2. Investigation of Molecular Interactions of Malvidin and Cyanidin on Various Biological Targets

Using the SwissTargetPrediction [27] server, a search for the affinity of biological targets with 2D and 3D similarity of malvidin and cyanidin profiles was performed, confronted with the library of active compounds and proteins available in the tool, simulating two species (*Homo sapiens* and *Mus musculus*). The server showed that proteins were among the best-ranked biological targets listed and were related to antioxidant mechanisms. Since the anthocyanidins showed biological activity for the proteins superoxide dismutase (SOD), catalase (CAT), reduced glutathione (GSH), and myeloperoxidase (MPO) in this study, the technique of blind molecular docking was applied to investigate molecular interactions, first calculating the docking of molecules by the protein surface to find the points with good binding affinities using the Achilles Blind Docking Server [37]. The information described in this work on the molecular interactions occurring between the anthocyanidins (malvidin and cyanidin) and the SOD, CAT, GSH MPO proteins may guide future in vitro/biochemical investigations. The proteins selected for the study are possible biological targets for the molecules malvidin and cyanidin, and the regions called binding sites to represent the most likely sites for the molecular interactions necessary for these couplings to occur. The main molecular interactions detected for the biological target SOD, including hydrophobic contacts, hydrogen bonds, and pi-stacking interactions are described in Table S4 (Supplementary Material), while detailed information about the binding affinity energy values (kcal/mol), number of generated conformations, poses, and the cluster coordinates are in Table S5. This information for the CAT target, is in Tables S6 and S7, for GSH in Tables S8 and S9, and for MPO Tables S10 and S11 (Supplementary Material).

#### 3.2.3. Superoxide Dismutase as a Biological Target

**Figure 11:**
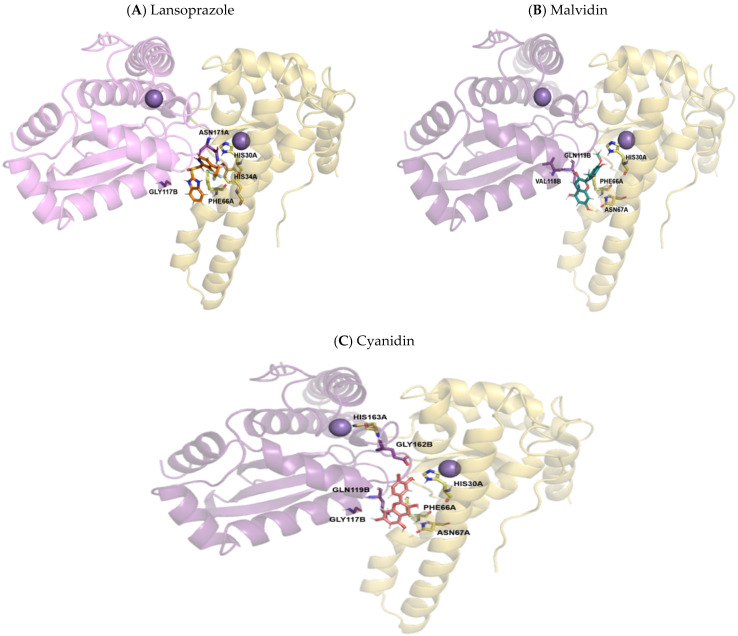
Molecular coupling of the lansoprazole (**A**), malvidin (**B**) and cyanidin (**C**) molecule in the Superoxide dismutase (SOD) protein structure–PDB 1N0J, between the A and B chains. (**A**) **Lansoprazole** molecule colored in orange, best pose with −7.2 kcal/mol (affinity energy), Mn^2+^ ion shown colored in purple ball. (**B**) **Malvidin:** Best pose with −7.5 kcal/mol (affinity energy), Mn^2+^ ion shown colored in purple ball, malvidin molecule colored in bluish-green. (**C**) **Cyanidin:** Best pose with −8.6 kcal/mol (affinity energy), Mn^2+^ ion shown colored in purple ball, cyanidin molecule colored in pink.

The three compounds act in very close coordinates, that is, they act practically on the same binding site, differing in only a few contacts. The chemical structure of lansoprazole was able to establish a halogen bond, which did not occur with the compounds malvidin and cyanidin. The reduction in gastric lesions observed in this study is possibly related to the binding site identified for the protein SOD, which is capable of satisfactorily coupling the tested anthocyanidins. Representations of the most significant structural details of this system can be seen in Figure 11.

#### 3.2.4. Catalase as a Biological Target

**Figure 12:**
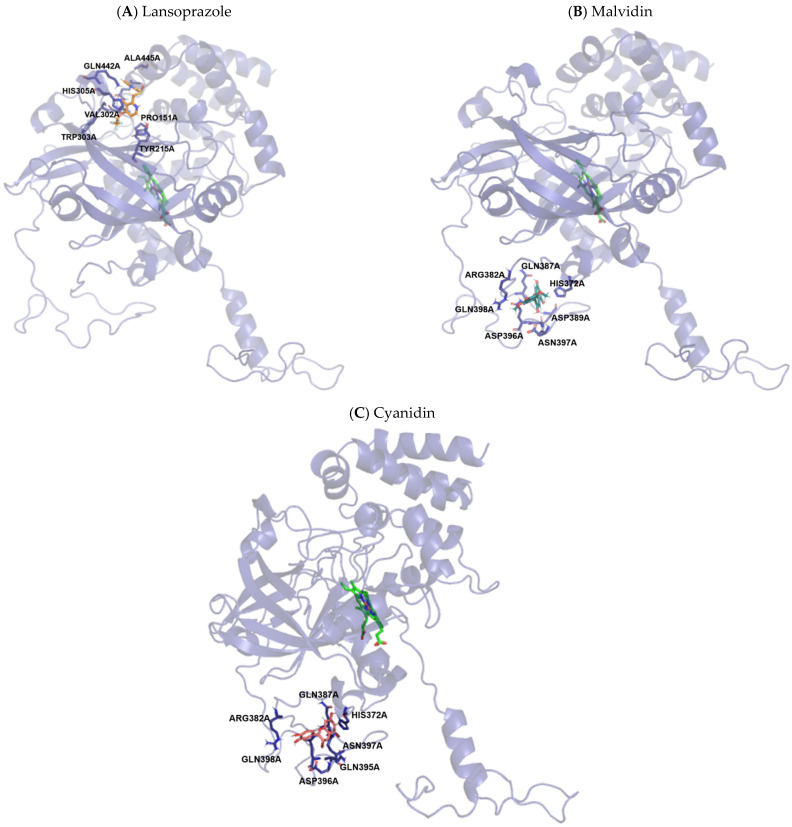
Molecular coupling of the lansoprazole (**A**), malvidin (**B**) and cyanidin (**C**) molecules in the Catalase (CAT) protein structure–PDB 1F4J, docking in chain A. (**A**) **Lansoprazole**: Best pose with −7.7 kcal/mol (affinity energy). Heme group shown colored in green. Lansoprazole molecule colored in orange. (**B**) **Malvidin**: Best pose with −7.6 kcal/mol (affinity energy). Heme group shown colored in green. Malvidin molecule colored in bluish-green. (**C**) **Cyanidin:** Best pose with −7.9 kcal/mol (affinity energy). Heme group shown colored in green. Cyanidin molecule colored in pink.

Analysis of the possible binding sites for CAT revealed that lansoprazole binds in a different region and far from the affinity sites of the two tested anthocyanidins, which might be related to the efficient response of these molecules observed in this study. As shown in Table S4, there are numerous molecular interactions, they do not involve residues important for inhibition, but involve residues that can increase CAT activity. Representations of the most significant structural details of this system can be seen in Figure 12.

#### 3.2.5. Reduced Glutathione as a Biological Target

**Figure 13:**
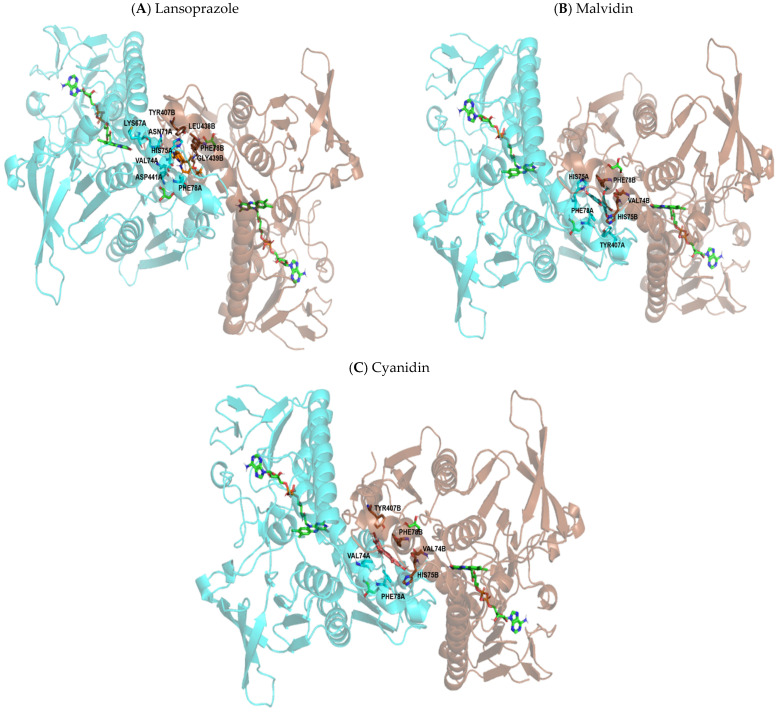
Molecular coupling of the lansoprazole (**A**), malvidin (**B**) and cyanidin (**C**) molecules in Glutathione reduced (GSH) protein structure–PDB 3SQP, docking in chain A and B. (**A**) **Lansoprazole**: Best pose with −9.0 kcal/mol (affinity energy). FAD and glycerol molecules shown colored in green. Lansoprazole molecule colored in orange. (**B**) **Malvidin**: Best pose with −8.5 kcal/mol (affinity energy). FAD and glycerol molecules shown colored in green. Malvidin molecule colored in bluish-green. (**C**) **Cyanidin:** Best pose with −8.9 kcal/mol (affinity energy). FAD and glycerol molecules shown colored in green. Cyanidin molecule colored in pink.

Analyzing this biological system, a region of affinity was observed for all three compounds; lansoprazole establishes a salt-bridge-type interaction that does not occur in anthocyanidins. Malvidin treatment showed elevation of GSH level in the stomach, probably because it has an appropriate number of molecular interactions involving important residues, as shown Table S5. Representations of the most significant structural details of this system can be seen in Figure 13.

#### 3.2.6. Myeloperoxidase as a Biological Target

**Figure 14:**
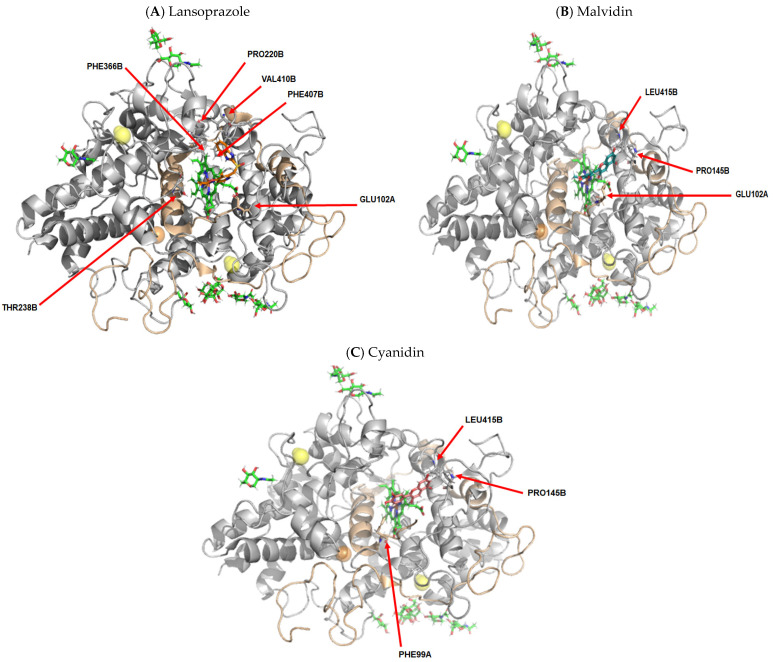
Molecular coupling of the lansoprazole (**A**), malvidin (**B**) and cyanidin (**C**) molecules on Myeloperoxidase (MPO) protein–PDB 7LAE, docking chain A and B. (**A**) **Lansoprazole**: Best pose with −7.5 kcal/mol (affinity energy). FAD and glycerol molecules shown colored in green. Mn^2+^ and Cl^-^ ion are shown colored in orange and yellow ball, respectively. Lansoprazole molecule colored in orange. (**B**) **Malvidin**: Best pose with −7.0 kcal/mol (affinity energy). FAD and glycerol molecules shown colored in green. Mn^2+^ and Cl^-^ ion are shown colored in orange and yellow ball, respectively. Malvidin molecule colored in bluish-green. (**C**) **Cyanidin:** Best pose with −7.6 kcal/mol (affinity energy). FAD and glycerol molecules shown colored in green. Mn^2+^ and Cl^-^ ion are shown colored in orange and yellow ball, respectively. Cyanidin molecule colored in pink.

Significant reduction in gastric lesions is attributed to anti-inflammatory action through myeloperoxidase interactions of the compound’s lansoprazole, malvidin, and cyanidin. The interaction may be related to the residual protein composition of a binding site near a heme group; although the three tested compounds did not establish direct molecular interactions with this group, this may be a key region of the protein. The main molecular interactions identified in this biological system are listed in Table S6. Representations of the most significant structural details of this system can be seen in Figure 14.

The docking study identifying the possible binding sites on the biological targets SOD, CAT, GSH, and MPO for coupling of the molecules lansoprazole, malvidin, and cyanidin, indicated the evaluation of molecular interactions profile being important for understanding the subtle differences that occur at the time of molecular coupling in each protein mentioned above. This is a preliminary study; the study results provide relevant information about the four biological systems studied, enabling the advancement of future complementary and in-depth studies on the interactions of these anthocyanidins in the human body.

## 4. Discussion

Alcohol consumption is associated with the development of gastric mucosal lesions in gastritis, gastric ulcers, and gastric carcinoma [38]. Chronic consumption of ethanol promotes gastric ulceration, decreases mucus production, reducing blood flow which further leads to injury at microvascular level, triggering an inflammatory process [39]. Ethanol-induced gastric damage involves tissue lipid peroxidation due to accumulation of reactive oxygen species and inflammation [40]. Ethanol-induced ulcer model is frequently used for characterizing new gastric protective agents, because ethanol predominantly affects the glandular portion of the stomach, increases lipid peroxidation, and decreases SOD activity, CAT activity, and GSH level [41]. Furthermore, ethanol depletes mucus from the gastric mucosal barrier [42], reduces gastric microcirculation [43] and inhibits the synthesis of prostaglandins [44], resulting in damage to the gastric mucosa. Gastrointestinal disorders related to alcohol consumption are critical in clinical gastroenterology because of the adverse effects caused by this agent [45].

In this study, we investigated the gastroprotective potential of cyanidin (C_15_H_11_O_6_•Cl), and malvidin chloride (C_17_H_15_O_7_•Cl) against the action of ethanol on the gastric mucosa in male and female mice (ovariectomized, with or without supplementation with 17-β estradiol). Several studies highlighted the growing need to include experimental group of females to elucidate the biological differences between the sexes, differences in drug tolerance, and differences in the course of disease and its treatment in a clear and coherent way [6,46,47,48]. Some studies reported that differences in the incidence rate of the disease between sexes are related to sex hormones, and that female hormones exert a protective effect on ulceration [49,50]. Thus, similar to other organs, estrogen may have a protective effect on the gastrointestinal tract [51]. Studies have demonstrated that sex hormones modulate GI motility, intestinal permeability, and barrier function, encompassing mucosal immunity [52]. Thus, pretreatment with cyanidin and malvidin in absolute ethanol-induced gastric ulcer model showed the best response in the male group at a dose of 5 mg·kg^−1^ for the inhibition of gastric damage in both compounds. In ovariectomized and supplemented females, malvidin treatment resulted in 90.8% fewer lesions compared to the control group, at the same dose as male animals (5 mg·kg^−1^), whereas cyanidin showed an inhibition of 80.7% at a dose of 20 mg·kg^−1^. Therefore, malvidin had higher gastroprotective effect than that of cyanidin at four-times lower dose in ovariectomized and estradiol-supplemented females. On the other hand, in ovariectomized animals, only a non-significant decrease was observed at doses of 5 mg·kg^−1^ and 10 mg·kg^−1^ (malvidin and cyanidin, respectively) in the macroscopic analysis of the gastric lesion.

Ethanol-induced neutrophil infiltration in the gastric mucosa has been shown to be closely related to the formation of lesions [53]. Determination of MPO activity, the main marker of neutrophil infiltration, is considered one of the most valid markers of acute injury [54]. Invasion of gastric tissues by neutrophils, marked with increased MPO activity, causes damage to the gastric mucosa [55]. These formations trigger the release of other pro-inflammatory mediators and the inflammatory cascade by increasing neutrophil invasion, accelerating gastric damage [55,56]. Malvidin treatment at a dose of 5 mg·kg^−1^ resulted in reduction in macroscopic lesions accompanied by a reduction in MPO activity in males and ovariectomized and supplemented groups (male, female ovariectomized supplemented or not); the animals subjected to ovariectomy receiving malvidin also showed a reduction in MPO activity but no macroscopic reduction. The same result was observed for this group of female animals treated with cyanidin at a dose of 10 mg·kg^−1^. The reduction in lesion formation in animals treated with cyanidin at a dose of 20 mg·kg^−1^ (ovariectomized and supplemented) and 5 mg·kg^−1^ (males) was accompanied by a reduction in MPO activity. According to in silico results, the anti-inflammatory activity (MPO) of the compounds can be related to the residual protein composition of a binding site, residue GLU102 and PHE99, respectively, for malvidin and cyanidin.

Ethanol consumption can significantly stimulate ROS generation and promote lipid peroxidation, followed by multiple gastric mucosal injuries. The gastrointestinal tract has enzymatic and non-enzymatic defense barriers against ROS [57]. Superoxide dismutase removes superoxide anions from the cellular environment by converting them to hydrogen peroxide or hydroxyl radicals. Increased SOD activity repairs gastric ulcers [58,59]. Ovariectomized and malvidin-treated female mice showed a significant difference in SOD activity.

The most efficient component of the antioxidant system is CAT, which can scavenge ROS by catalyzing the decomposition of H_2_O_2_ into water and oxygen [60]. Catalase activity was elevated in male animals treated with cyanidin (5 mg·kg^−1^). Glutathione is an endogenous antioxidant; its properties are related to the presence of a thiol group in its chemical structure [61,62]; it acts as an antioxidant by directly scavenging free radicals or by acting as a cofactor for antioxidant enzymes [63,64]. It is known that ethanol administration promotes an increase in ROS level and a reduction in GSH level in the gastric mucosa [65]. An increase in GSH level was observed in the mucosa of ovariectomized females treated with malvidin. Regarding cyanidin (20 mg·kg^−1^), ovariectomized females supplemented with estradiol showed an increase in GSH level compared to the vehicle group.

This study investigated the response of the two anthocyanidins against the harmful effects of ethanol. Both malvidin and cyanidin showed gastroprotection; this effect was accompanied by changes in antioxidant and anti-inflammatory parameters. Malvidin showed gastroprotective activity independent of the hormonal status of animals and was effective at a dose of 5 mg·kg^−1^. However, the difference observed was with respect to the mechanisms of gastroprotection. The same dose that was effective in preventing the formation of lesions in males was useful in promoting lesion inhibition in females, supplemented and not supplemented. Despite this, the mechanisms of gastroprotection differed among males (reduced MPO), supplemented females (reduced MPO), and non-supplemented females (reduced MPO and increased GSH).

In contrast, cyanidin was efficient at different doses for the three hormonal scenarios investigated, with different mechanisms of action for this model. Furthermore, the mechanisms of gastroprotection differed between males (reduced MPO) and supplemented and non-supplemented females (reduced MPO and increased GSH), indicating that the antioxidant system play more important role in females than in males treated with cyanidin. Similar to malvidin, the male group did not show any activity on antioxidant enzymes, whereas the female group showed an increase in GSH and SOD. Furthermore, estrogen appears to be directly linked to the antioxidant system, as estrogen activates antioxidant enzymes and decreases ROS [66]. In a study by Okada, it was found that men aged >70 years and postmenopausal women had a higher incidence of peptic ulcer than the others, demonstrating that a decrease in serum estrogen level at this age and stage of individuals decreases the production of mucus in the stomach [67]. Another study showed that, in women aged >50 years, the decreasing level of estrogen in the body directly interfered with few diseases of the gastrointestinal tract. For instance, women of this age or older had a higher prevalence and severity of gastroesophageal reflux than the others [68]. Estrogen has been shown to have anti-inflammatory function and can delay the development of gastroesophageal reflux [69]. Studies have shown that estrogen has an anti-ulcer activity, because it maintains the integrity of the mucus in the stomach wall and has a protective effect, due to its antioxidant properties, vasodilator action, and increased angiogenesis [70,71]. These two mechanisms of action are directly linked to gastric ulcerations caused by ethanol; therefore, the increased activity of antioxidant enzymes in females supplemented with 17β-estradiol (500 µg/kg) receiving pretreatment with anthocyanidins can be explained.

In silico analysis helped us to understand how conformational differences between malvidin and cyanidin molecules could possibly act on biological targets: SOD, CAT, GSH and MPO, which clarified our understanding of gastroprotection mechanisms and in vivo differences in treatments observed in this study. These assays must be validated and confirmed by other analyses, but they collaborate with the gain of information about the pharmacological activity of possible therapeutic targets. Although cyanidin and malvidin molecules have a similar chemical structure, and the same dose is able to promote the inhibition of ethanol-induced gastric lesions in males, there is need to study the mechanisms of gastroprotection in other biological systems under different situations such as in females, with or without hormonal supplementation. The results of this study support the conclusion of the study by Hureau [52], which indicated that it is necessary to consider the differences in hormonal status to obtain a better understanding of the gastrointestinal physiology. Additionally, one must consider the differences in hormonal status when evaluating the potential of new therapeutic targets.

## 5. Conclusions

In conclusion, our study provides evidence for the pharmacological effects of anthocyanidins in peptic ulcer disease. The pharmacological activity was observed to be associated with the reduction in inflammatory biomarkers such as MPO, and the triggering of the endogenous antioxidant defense system such as CAT, SOD, and GSH. The initial analysis of association between ethanol-induced ulceration and different hormonal status verified that malvidin presents gastroprotection, regardless of the hormonal action and variations in males and females. Cyanidin has gastroprotective activity; however, the effective dose in males differs from that in females under different hormonal situations (presence or absence of supplementation with estradiol), thus, indicating that the molecule possesses hormone-dependent activity, which should be further investigated. The in silico analyses showed, for the first time, the possible binding site in selected targets that contributes to gastroprotection, and opened a new landscape that should be further validated with additional biochemical and in vitro experiments.

## Data Availability

The data presented in this study are available on request from the corresponding author.

## Supplementary Materials

The following supporting information can be downloaded at: https://www.mdpi.com/article/10.3390/pharmaceutics14030565/s1, Figure S1: Plasma concentrations of estradiol quantified by radioimmune assay of ovariectomized and intact females. We can observe that intact animals have a higher concentration of total estradiol, when compared to the ovariectomized (OVX) females; Figure S2: Vaginal smear realized in the animals submitted to ethanol induced gastric ulcer re-alized in the 29th day of experimental calendar (a) groups of female animals ovariectomized and not supplemented (b) group of females ovariectomized and supplement with 17-β-estradiol (c) intact females. Magnification of 400x [72,73,74]; Figure S3: Structure of Superoxide dismutase (SOD) protein - PDB 1N0J, docking in chain A and B, where the representations: A, B and C, correspond respectively to the mapping of affinity sites of the molecules Lansoprazole, Malvidin and Cyanidin. Mn^2+^ ion shown col-ored in purple ball. Figure S4: Structure of Catalase (CAT) protein - PDB 1F4J, docking in chain A, where the representations: A, B, and C, correspond respectively to the affinity site mapping of the molecules Lansoprazole, Malvidin, and Cyanidin. Heme group shown colored in green; Figure S5: Structure of Glutathione reductase (GSH) protein - PDB 3SQP, docking chain A and B, where the representations: A, B, and C, correspond respectively to the affinity site mapping of Lansoprazole, Malvidin, and Cyanidin molecules. FAD and glycerol molecules shown colored in green; Figure S6: Structure of Myeloperoxidase (MPO) protein - PDB 7LAE, where the representa-tions: A, B, and C, correspond respectively to the affinity site mapping of the molecules Lansoprazole, Malvidin, and Cyanidin. Cl^−^ and Ca^2+^ ion represented as a yellow and or-ange colored ball respectivel; heme group; acetamido-2-deoxy-beta-D-glucopyranose; be-ta-D-manopyranose; alpha-D-manopyranose; and alpha-L-fucopyranose shown colored in green; Table S1: Effect of oral treatment with malvidin or cyanidin chloride in the prevention of gastric lesions induced by ethanol (results presented as: mean ± standard error of the mean); Table S2: Prediction of ADMET properties by pkCSM server for the molecules Lansoprazole, Malvidin and Cyanidin; Table S3: Analysis of in silico toxicity of malvidin and cyanidin (Prediction of ADMET); Table S4: Main molecular interactions detected in the protein (SOD); Table S6: Major molecular interactions detected in the protein (CAT); Table S7: Details of Catalase (CAT) binding site mapping; Table S8: Major molecular interactions detected in the protein (GSH); Table S9: Mapping details of Glutathione reductase (GSH) binding sites; Table S10: Main molecular interactions detected in the Myeloperoxidase (MPO) protein; Table S11: Mapping details of Myeloperoxidase (MPO) binding sites.
