# Supplementary material for: Relationship between Hormonal Modulation and Gastroprotective Activity of Malvidin and Cyanidin Chloride: In Vivo and In Silico Approach
Source: Pharmaceutics. 2022 Mar 4;14(3):565. doi: 10.3390/pharmaceutics14030565 (PMC8953580; doi:10.3390/pharmaceutics14030565)
Supplement: Supplementary file 1 [file pharmaceutics-14-00565-s001.zip › pharmaceutics-1571590-supplementary.pdf]

Table S1. Effect of oral treatment with malvidin or cyanidin chloride in the prevention of gastric lesions induced by ethanol (results presented as mean ± standard error of the mean).

Experimental Group	Treatment (p.o.)	Dose (mg/kg)	Lesion Area (mm^2)	MPO (Unit of MPO/g)	GSH (nmol/g)	CAT (Unit of CAT/g)	SOD (Unit of SOD/g)
Males	Vehicle	-	278.1 ± 68.9	39.2 ± 3.7	715.2 ± 64.3	37.64 ± 4.2	16.96 ± 2.5
	Lansoprazole	30	50.69 ± 25.8**	14.86 ± 1.3**	1016.0 ± 86.1*	62.18 ± 6.3**	23.24 ± 3.5
	Malvidin	2.5	199.1 ± 78.3	26.77 ± 2.8	-	-	-
		5	25.31 ± 8.7**	16.26 ± 2.3*	627.5 ± 77.7	48.33 ± 4.2	11.27 ± 1.7
		10	70.26 ± 57.5*	36.27 ± 13.7	-	-	-
	Cyanidin	5	71.16 ± 33.9*	19.29 ± 4.2*	811.2 ± 32.4	58.92 ± 5.4**	8.51 ± 1.3
		10	49.81 ± 18.8**	21.56 ± 5.1	-	-	-
		20	48.33 ± 4.8**	15.77 ± 3.9*	-	-	-
	Not treated	-	-	16.8 ± 1.6**	1156.0 ± 85.5*	57.0 ± 1.4*	28.33 ± 2.8*
Females - ovariecto-mized andsupple-mented	Vehicle	-	235.2 ± 55.6	19.17 ± 1.9	72.48 ± 6.7	61.02 ± 5.9	5.49 ± 0.5
	Lansoprazole	30	75.99 ± 14.4*	12.07 ± 0.2**	120.1 ± 8.7**	87.86 ± 13.9	7.12 ± 1.6
	Malvidin	2.5	93.27 ± 35.6	14.07 ± 1.2	-	-	-
		5	21.51 ± 8.8*	11.95 ± 0.4**	98.18 ± 13.4	67.16 ± 10.7	5.53 ± 1.2
		10	209.1 ± 70.6	16.21 ± 1.4	-	-	-
	Cyanidin	5	162.0 ± 78.4	14.62 ± 1.7	-	-	-
		10	132.9 ± 29.8	15.19 ± 1.2	-	-	-
		20	45.37 ± 19.9*	12.36 ± 0.8**	109.3 ± 9.9*	63.10 ± 4.5	7.0 ± 0.9
	Not treated	-	-	10.84 ± 0.7***	103.9 ± 3.8	90.15 ± 26.9	7.4 ± 1.4
Females - ovariecto-mized	Vehicle	-	129.1 ± 44.3	16.98 ± 1.4	69.01 ± 11.4	60.48 ± 10.9	5.45 ± 1.3
	Lansoprazole	30	38.28 ± 15.8	11.04 ± 0.7	89.1 ± 12.8	75.20 ± 8.8	8.4 ± 0.7
	Malvidin	2.5	285.6 ± 124.1	13.28 ± 1.6	-	-	-
		5	48.63 ± 12.4	9.24 ± 1.7**	128.2 ± 10.1*	58.10 ± 5.1	11.06 ± 1.6*
		10	443.5 ± 111.7	16.53 ± 2.3	-	-	-
	Cyanidin	5	218.8 ± 109.2	9.1 ± 2.1**	-	-	-
		10	18.8 ± 11.1	10.62 ± 0.5	116.0 ± 18.7	74.96 ± 11.2	5.92 ± 1.2
		20	61.35 ± 29.4	12.07 ± 1.3	-	-	-
	Not treated	-	-	11.88 ± 0.4	92.67 ± 2.9	108.1 ± 10.9*	7.77 ± 0.9

Data are presented as mean ± S.E.M. ANOVA followed by the Dunnett's test; where * $p < 0.05$ and ** $p < 0.01$, compared to the respective vehicle group.

1. Hormonal blockade by surgical method

To investigate the effect of ovarectomization in female mice was realized the quantification of estradiol in plasma samples thought the radioimmune assay. We collected samples by cardiac puncture from ovariectomized females (OVX) and intact animals, that did not pass by a surgical process. We can observe that estradiol levels of intact animals are higher, when compared to the ovariectomized group (OXV), showing that the surgical procedure was capable to reduce the circulating levels of estradiol (see in Figure S1).

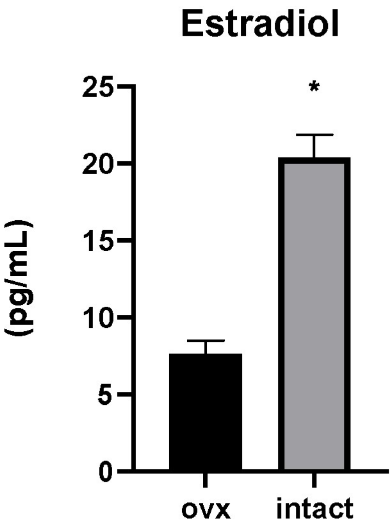

Figure S1. Plasma concentrations of estradiol quantified by radioimmune assay of ovariectomized and intact females. We can observe that intact animals have a higher concentration of total estradiol, when compared to the ovariectomized (OVX) females. Data are presented as mean ± S.E.M. ANOVA followed by the Dunnett's test; where * $p < 0.05$, compared to the respective vehicle group.

2. Vaginal smear

The vaginal smear was done according to procedure of [1] , in the day of gastric ulcer induced by absolute ethanol, we analyze the vaginal washed content of females. The samples were inserted in glass slides and analyzed using an optical microscope in the magnification of 400x. In the vaginal smear, we can observe that ovariectomization implicated in the absence of epithelial cells (superficial and intermediate) (see in Figure S2a), but we can observe the presence of bacilli, that are common microbiota present in the vaginal region. In the OXV females followed by hormonal supplementation with 17-β-estradiol the results indicate a cellular profile fount in the normal vaginal mucosa, i.e., superficial cells suggesting the estrogenic phase (small nuclei) and intermediate cells where the nuclei are large (see in Figure S2b). In the intact females we analyze 4 animals and was possibly to note that each female was in a different phase of estrous cycle. Animals 1 and 2 has a large amount of superficial and a minor number of intermediate cells, but the animal number 3 and 4 have total absence of cells, such as the OVX females (see in Figure S2c). Based in this significant variation of estrous phase, we decide to use 17-β-estradiol as a pharmacological tool to normalize the hormonal levels and avoid the interference in the anthocyanidin treatment [2,3].

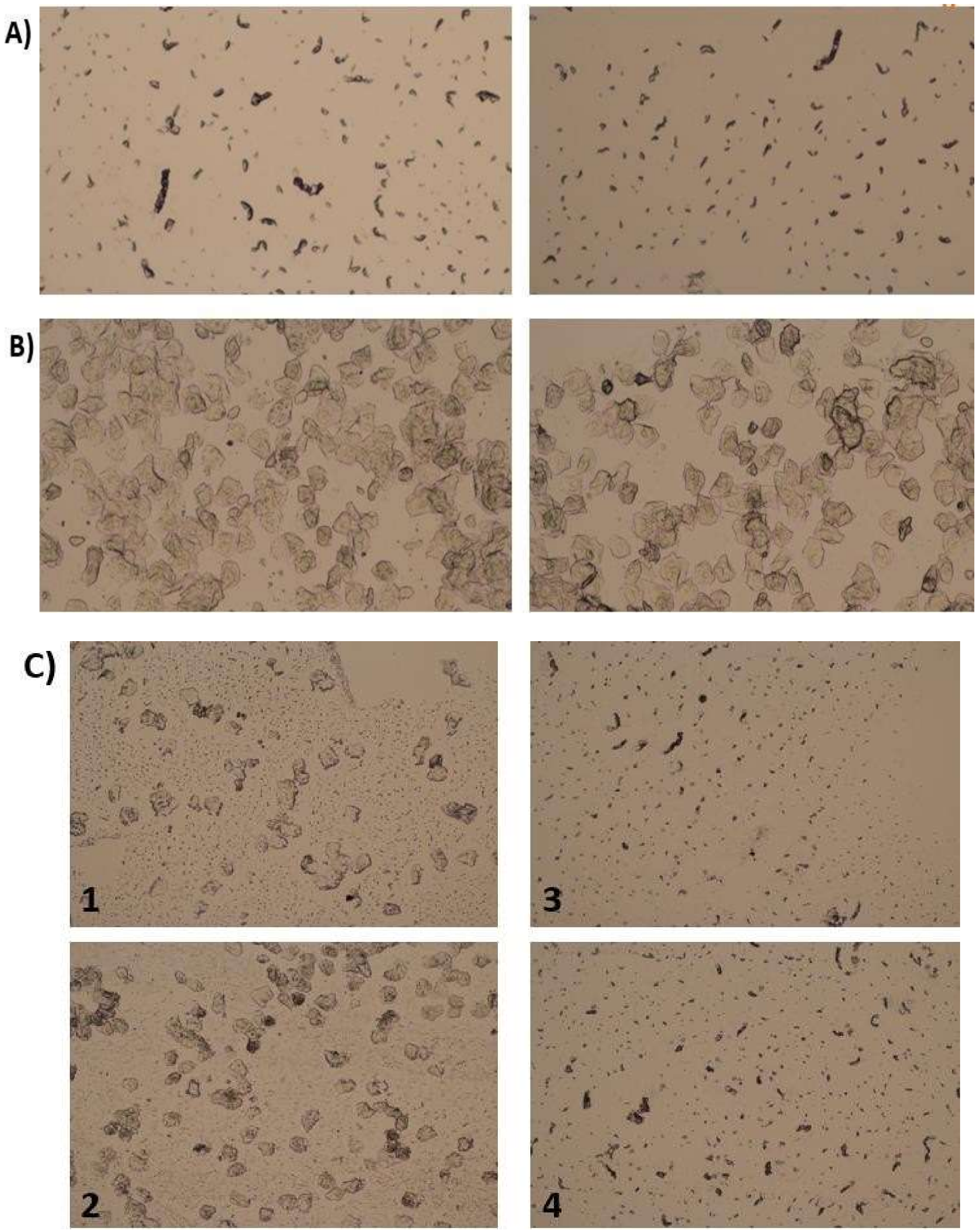

Figure S2. Vaginal smear realized in the animals submitted to ethanol induced gastric ulcer realized in the 29th day of experimental calendar (**A**) groups of female animals ovariectomized and not supplemented (**B**) group of females ovariectomized and supplement with 17-β-estradiol (**C**) intact females. Magnification of 400x.

Table S2. Prediction of ADMET properties by pkCSM server for the molecules Lansoprazole, Malvidin and Cyanidin.

Property	Model Name	Predicted Value-Lansoprazole	Predicted Value-Malvidin	Predicted Value-Cyanidin	Unit
**Absorption**	Water solubility	−2.767	−3.557	−3.129	Numeric (log mol/L)
**Absorption**	Caco2 permeability	1.055	−0.088	−0.357	Numeric (log Papp in 10^{-6} cm/s)
**Absorption**	Intestinal absorption (human)	84	59.362	67.885	Numeric (% Absorbed)
**Absorption**	Skin Permeability	−2.735	−2.735	−2.735	Numeric (log Kp)
**Absorption**	P-glycoprotein substrate	Yes	Yes	Yes	Categorical (Yes/No)
**Absorption**	P-glycoprotein I inhibitor	No	No	No	Categorical (Yes/No)
**Absorption**	P-glycoprotein II inhibitor	Yes	No	No	Categorical (Yes/No)
**Distribution**	VDss (human)	0.377	0.377	0.086	Numeric (log L/kg)
**Distribution**	Fraction unbound (human)	0.051	0.061	0.090	Numeric (Fu)
**Distribution**	BBB permeability	−0.669	−1.09	−1.09	Numeric (log BB)
**Distribution**	CNS permeability	−2.946	−3.644	−2.957	Numeric (log PS)
**Metabolism**	CYP2D6 substrate	No	No	No	Categorical (Yes/No)
**Metabolism**	CYP3A4 substrate	No	No	No	Categorical (Yes/No)
**Metabolism**	CYP1A2 inhibitior	Yes	Yes	Yes	Categorical (Yes/No)
**Metabolism**	CYP2C19 inhibitior	Yes	No	No	Categorical (Yes/No)
**Metabolism**	CYP2C9 inhibitior	Yes	No	Yes	Categorical (Yes/No)
**Metabolism**	CYP2D6 inhibitior	No	No	No	Categorical (Yes/No)
**Metabolism**	CYP3A4 inhibitior	Yes	No	No	Categorical (Yes/No)
**Excretion**	Total Clearance	0.662	0.922	0.763	Numeric (log ml/min/kg)
**Excretion**	Renal OCT2 substrate	Yes	No	No	Categorical (Yes/No)
**Toxicity**	AMES toxicity	Yes	No	No	Categorical (Yes/No)
**Toxicity**	Max. tolerated dose (human)	0.238	0.954	0.794	Numeric (log mg/kg/day)
**Toxicity**	hERG I inhibitor	No	No	No	Categorical (Yes/No)
**Toxicity**	hERG II inhibitor	No	Yes	No	Categorical (Yes/No)
**Toxicity**	Oral Rat Acute Toxicity (LD50)	2.523	1.933	2.371	Numeric (mol/kg)
**Toxicity**	Oral Rat Chronic Toxicity (LOAEL)	1.645	2.338	2.022	Numeric (log mg/kg_bw/day)
**Toxicity**	Hepatotoxicity	Yes	No	No	Categorical (Yes/No)
**Toxicity**	Skin Sensitisation	No	No	No	Categorical (Yes/No)
**Toxicity**	*T.Pyriformis*toxicity	0.285	0.306	0.302	Numeric (log ug/L)
**Toxicity**	Minnow toxicity	1.212	0.982	1.795	Numeric (log mM)

Table S3. Analysis of in silico toxicity of malvidin and cyanidin (Prediction of ADMET).

**TOXICITY**				
	Cyanidin		***Malvidin***	
Human Ether-a-go-go-Related Gene	Weak inhibitor	0.9508	Weak inhibitor	0.9620
Inhibition	Non-inhibitor	0.8062	Non-inhibitor	0.8114
AMES Toxicity	Non AMES toxic	0.8841	Non AMES toxic	0.9211
Carcinogens	Non-carcinogens	0.9044	Non-carcinogens	0.8868
Fish Toxicity	High FHMT	0.9381	High FHMT	0.8027
Tetrahymena Pyriformis Toxicity	High TPT	0.9938	High TPT	0.9874
Honey Bee Toxicity	High HBT	0.6271	High HBT	0.7023

Biodegradation	Not ready biodegradable	0.9082	Not ready biodegradable	0.9561
Acute Oral Toxicity	II	0.5617	III	0.5653
Carcinogenicity (Three-class)	Non-required	0.5895	Non-required	0.5696

Toxicity result from the in silico prediction of the compounds malvidin and cyanidin. The lmmd platform was used and using the smile of each compound (respectively: COC1=CC(=CC(=C1O)OC)C2=[O+]C3=CC(=CC(=C3C=C2O)O)O.[Cl-] and C1=CC(=C(C=C1C2=[O+]C3=CC(=CC(=C3C=C2O)O)O)O)O.[Cl-]).

Superoxide dismutase (SOD)

Representations of the most significant structural details of this system.

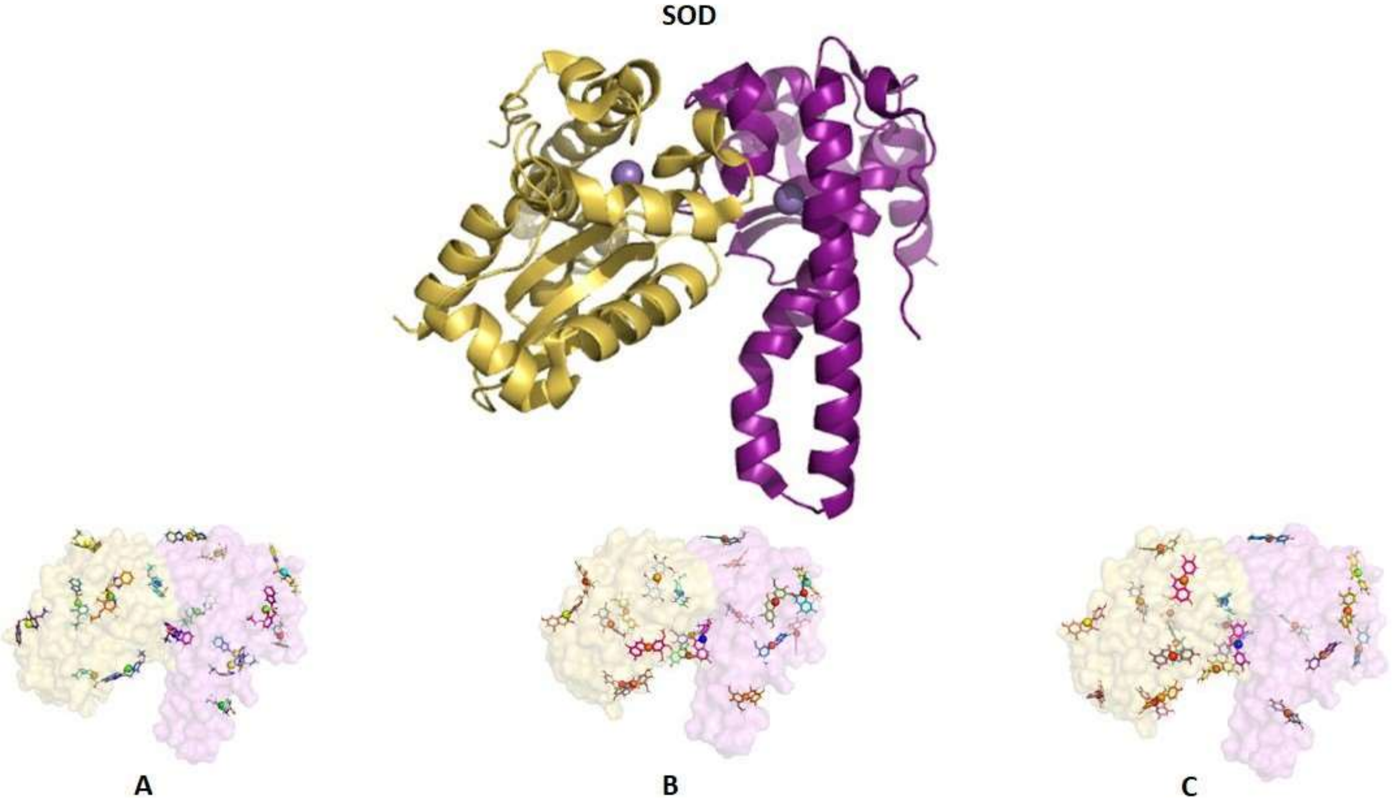

Figure S3. Structure of Superoxide dismutase (SOD) protein - PDB 1N0J, docking in chain A and B, where the representations: **A**, **B** and **C**, correspond respectively to the mapping of affinity sites of the molecules Lansoprazole, Malvidin and Cyanidin. Mn^{2+} ion shown colored in purple ball.

Table S4. Main molecular interactions detected in the protein (SOD).

Compound	Residue	Chain	Interaction
**lansoprazole**	TYR 34	A	hydrophobic contact
	GLN 119	B	
	ASN 171	B	
	HIS 30	A	hydrogen bonding
	GLY 117	B	
	PHE 66	A	pi-stacking
	PHE 66	A	
	ASN 171	B	halogen bonding
**malvidin**	GLN 119	B	hydrophobic contact
	HIS30	A	hydrogen bonding

	HIS 30	A	
	ASN 67	A	
	VAL 118	B	
	PHE 66	A	pi-stacking
**cyanidin**	PHE 66	A	hydrophobic contact
	GLN 119	B	
	HIS 30	A	hydrogen bonding
	ASN 67	A	
	GLY 117	B	
	GLU 162	B	
	HIS 163	A	
	PHE 66	A	pi-stacking

Table S5. Details of Superoxide dismutase (SOD) protein binding site mapping.

**Superoxide dismutase (SOD) - molecule Lansoprazole**				
**Cluster**	**affinity energy (kcal/mol)**	**nº ofposes**	**bestpose**	**site**
1	−7.2	87	362	16.08, −8.56, 40.91
2	−7.1	85	270	20.62, −1.21, 61.95
3	−6.6	44	388	14.90, −34.42, 60.74
4	−6.2	18	55	5.70, −0.36, 26.01
5	−6.2	22	251	3.58, −1.88, 74.61
6	−6.1	24	109	10.68, 10.58, 54.36
7	−6.0	13	14	34.75, 7.67, 53.80
8	−6.0	13	325	4.81, −14.72, 46.88
9	−5.9	43	127	18.25, 10.51, 33.13
10	−5.9	9	273	17.11, −22.54, 68.91
11	−5.9	11	301	14.47, −26.98, 42.38
12	−5.8	13	181	27.50, 24.91, 39.27
13	−5.7	5	350	5.24, −10.33, 67.58
14	−5.7	2	327	−4.68, −16.86, 58.41
15	−5.6	3	11	31.09, 1.25, 30.44
16	−5.5	2	213	28.08, −19.37, 45.12
17	−5.3	1	196	2.88, 16.97, 43.25
18	−4.6	1	394	−6.62, −29.77, 60.61
**Superoxide dismutase (SOD) - molecule Malvidin**				
**Cluster**	**affinity energy (kcal/mol)**	**nº ofposes**	**bestpose**	**site**
1	−7.5	87	68	14.46, −8.44, 40.64
2	−7.4	83	116	16.84, 0.80, 60.76
3	−6.7	43	378	13.34, −34.24, 62.17
4	−6.2	36	85	29.02, 24.49, 40.70
5	−5.8	6	18	35.30, 5.69, 53.63
6	−5.7	10	57	5.84, −5.10, 47.37
7	−5.7	18	244	1.32, −2.15, 70.86
8	−5.6	2	149	7.60, 1.43, 54.24
9	−5.5	3	215	28.14, −20.82, 46.63
10	−5.5	11	125	28.14, −20.82, 46.63

11	−5.5	11	305	14.85, −26.77, 42.46
12	−5.5	6	293	1.22, −22.44, 46.53
13	−5.4	15	128	13.25, 10.79, 34.87
14	−5.4	11	326	6.51, −15.96, 66.60
15	−5.4	13	49	5.28, 0.29, 25.20
16	−5.3	2	220	25.38, −13.80, 71.83
17	−5.3	6	274	17.74, −26.98, 68.04
18	−5.3	4	95	11.24, 19.16, 54.94
19	−5.2	11	12	28.14, 13.44, 32.76
20	−5.2	6	394	−4.78, −31.93, 59.19
21	−5.1	12	99	−4.78, −31.93, 59.19
**Superoxide dismutase (SOD) – molecule Cyanidin**				
**Cluster**	**affinityenergy (kcal/mol)**	**nº ofposes**	**bestpose**	**site**
1	−8.6	96	160	14.83, −8.11, 41.47
2	−8.3	86	119	17.14, 0.55, 59.81
3	−7.0	44	389	16.33, −34.50, 59.70
4	−6.5	37	183	28.61, 24.97, 40.49
5	−6.2	18	77	20.13, 8.60, 31.68
6	−6.2	7	54	6.29, −5.76, 45.95
7	−6.1	17	135	11.29, 19.59, 54.58
8	−6.1	8	305	1.08, −22.69, 46.83
9	−6.1	19	297	−4.69, −31.25, 60.22
10	−6.0	6	20	35.09, 6.07, 53.73
11	−6.0	4	148	9.66, 4.84, 54.52
12	−6.0	9	351	6.95, −16.25, 66.72
13	−6.0	15	243	1.07, −1.91, 70.12
14	−5.9	2	216	28.32, −20.73, 46.76
15	−5.9	5	220	17.16, −22.73, 69.60
16	−5.7	4	9	30.20, −0.62, 31.14
17	−5.7	9	48	6.32, 4.35, 34.96
18	−5.6	1	104	23.86, 15.98, 59.65
19	−5.6	6	47	6.82, −1.14, 24.03
20	−5.4	1	196	2.32, 17.96, 44.79
21	−5.3	1	197	8.68, 28.29, 39.38
22	−5.3	1	51	0.52, −11.57, 32.15

Catalase (CAT)

Representations of the most significant structural details of this system.

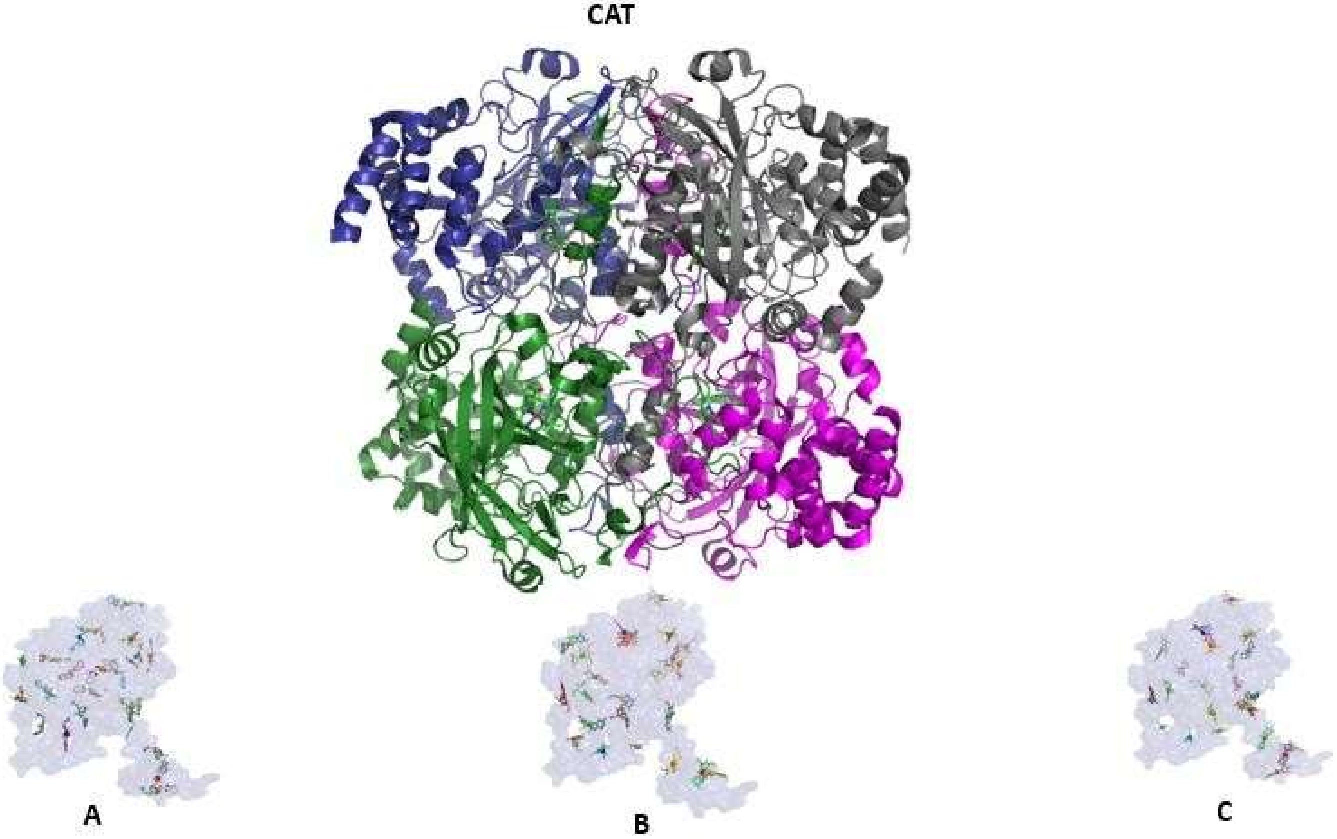

Figure S4. Structure of Catalase (CAT) protein - PDB 1F4J, docking in chain A, where the representations: **A**, **B**, and **C**, correspond respectively to the affinity site mapping of the molecules Lansoprazole, Malvidin, and Cyanidin. Heme group shown colored in green.

Table S6. Major molecular interactions detected in the protein (CAT).

Compound	residue	chain	interaction
**lansoprazole**	PRO 151	A	hydrophobic contact
	TYR 215	A	
	VAL 302	A	
	GLN 442	A	
	ALA 445	A	
	TRP 303	A	hydrogen bonding
	LYS 306	A	
	HIS 305	A	pi-stacking
**malvidin**	GLN 387	A	hydrophobic contact
	ARG 382	A	hydrogen bonding
	ARG 382	A	
	ASP 389	A	
	ASP 396	A	
	ASP 396	A	
	ASN 397	A	
	GLN 398	A	
	GLN 398	A	
	HIS 372	A	pi-stacking
**cyanidin**	GLN 387	A	hydrophobic contact
	ARG 382		hydrogen bonding
	GLN 387	A	

	GLN 395	A	
	ASP 396	A	
	ASN 397	A	
	GLN 398	A	
	GLN 398	A	
	HIS 372	A	pi-stacking

Table S7. Details of Catalase (CAT) binding site mapping.

Catalase (CAT) – molecule Lansoprazole				
**Cluster**	**affinity energy (kcal/mol)**	**nº ofposes**	**bestpose**	**site**
1	−7.7	90	211	63.32, 77.67, 4.88
2	−7.6	86	370	47.04, 81.47, 38.50
3	−7.2	71	98	56.62, 99.91, 3.96
4	−6.8	35	122	63.38, 93.60, 26.12
5	−6.8	37	47	39.21, 99.02, 22.66
6	−6.7	28	295	36.10, 76.25, 23.14
7	−6.6	30	406	74.45, 77.91, 21.02
8	−6.6	6	200	68.75, 63.09, 22.41
9	−6.5	11	166	68.64, 103.41, 17.42
10	−6.4	10	405	73.71, 87.09, 24.58
11	−6.4	2	187	50.90, 90.04, −4.83
12	−6.4	31	35	27.76, 109.66, 30.13
13	−6.3	8	464	68.10, 92.24, −12.26
14	−6.3	3	393	51.32, 72.22, 39.66
15	−6.2	8	458	79.33, 92.57, 6.86
16	−6.2	11	247	35.12, 93.63, 15.09
17	−6.0	1	72	64.03, 75.55, 30.58
18	−6.0	4	68	58.88, 62.88, 32.27
19	−5.7	3	9	19.72, 111.08, 39.82
20	−5.6	1	448	65.34, 112.18, 5.45
21	−5.6	6	253	38.32, 76.64, 4.68
22	−4.9	1	0	16.13, 104.60, 32.52
**Catalase (CAT) – molecule Malvidin**				
**Cluster**	**affinityenergy (kcal/mol)**	**nº ofposes**	**bestpose**	**site**
1	−7.6	65	352	43.60, 80.93, 41.65
2	−7.3	86	424	62.97, 79.53, 2.87
3	−6.5	24	399	74.97, 74.31, 20.01
4	−6.5	83	184	57.12, 100.14, 4.35
5	−6.5	15	45	41.14, 92.62, 35.63
6	−6.4	34	40	40.86, 99.17, 21.12
7	−6.3	22	321	74.47, 83.58, 26.37
8	−6.2	23	197	63.78, 75.77, 30.02
9	−6.2	9	113	49.12, 75.25, 35.35
10	−6.1	17	72	52.69, 61.75, 29.46
11	−6.1	7	218	51.99, 90.48, −4.61
12	−6.1	23	110	36.55, 77.72, 24.29
13	−5.9	17	457	79.51, 92.28, 7.11

14	−5.9	4	49	64.00, 92.65, 27.91
15	−5.8	4	450	69.15, 106.57, 14.45
16	−5.6	34	15	24.30, 115.47, 31.16
17	−5.5	1	67	64.07, 61.78, 13.52
18	−5.5	3	385	55.76, 76.74, 45.78
19	−5.3	4	464	69.93, 91.64, −12.38
20	−5.3	1	252	50.75, 74.02, 2.44
21	−5.3	4	251	38.40, 90.75, 0.70
22	−5.1	1	448	69.58, 112.86, 1.11
23	−5.0	1	1	28.48, 100.99, 33.14
24	−4.5	1	0	17.94, 105.83, 29.02
**Catalase (CAT) – molecule Cyanidin**				
**Cluster**	**affinityenergy (kcal/mol)**	**nº ofposes**	**bestpose**	**site**
1	−7.9	70	344	44.16, 81.28, 41.84
2	−7.7	100	256	63.47, 79.58, 2.58
3	−7.2	14	308	42.03, 92.50, 36.09
4	−6.9	26	407	73.80, 87.08, 25.38
5	−6.8	8	141	63.41, 92.67, 27.13
6	−6.8	69	90	57.28, 100.10, 4.57
7	−6.8	17	39	39.95, 99.86, 23.38
8	−6.7	34	391	61.76, 75.38, 30.61
9	−6.7	22	299	39.96, 97.38, 16.66
10	−6.6	3	149	57.71, 104.11, 20.84
11	−6.6	27	296	36.70, 78.43, 24.39
12	−6.5	19	76	52.55, 61.66, 28.93
13	−6.4	6	266	75.67, 74.07, 18.43
14	−6.3	17	163	79.34, 91.86, 6.95
15	−6.2	4	187	52.34, 90.78, −4.35
16	−6.0	2	450	69.04, 106.39, 15.09
17	−6.0	3	382	55.67, 75.94, 45.14
18	−5.9	1	249	29.76, 85.72, 16.08
19	−5.9	21	18	24.14, 115.36, 31.04
20	−5.9	13	10	20.22, 110.55, 40.43
21	−5.6	3	467	70.10, 91.25, −12.26
22	−5.5	1	448	72.28, 112.59, 1.84
23	−5.3	1	215	50.88, 75.12, 2.13
24	−5.3	1	1	29.63, 104.31, 30.23
25	−5.0	1	0	17.65, 105.68, 29.60

Glutathione reductase (GSH)

Representations of the most significant structural details of this system.

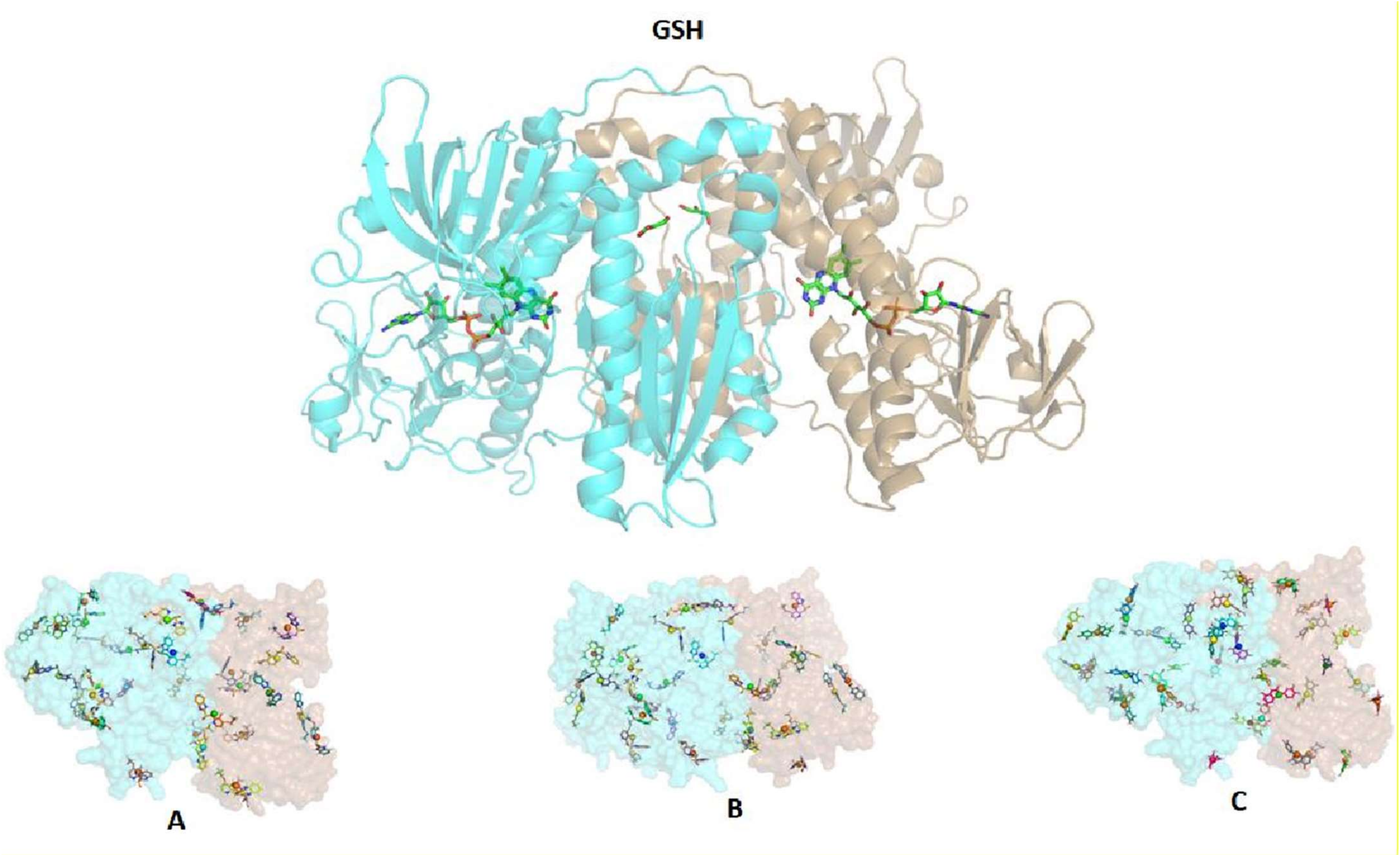

Figure S5. Structure of Glutathione reductase (GSH) protein - PDB 3SQP, docking chain A and B, where the representations: **A**, **B**, and **C**, correspond respectively to the affinity site mapping of Lansoprazole, Malvidin, and Cyanidin molecules. FAD and glycerol molecules shown colored in green.

Table S8. Major molecular interactions detected in the protein (GSH).

Compound	residue	chain	interaction
**lansoprazole**	VAL 74	A	hydrophobic contact
	PHE 78	A	
	PHE 78	A	
	PHE 78	A	
	TYR 407	B	
	LEU 438	B	
	LEU 438	B	
	LYS 67	A	hydrogen bonding
	ASN 71	A	
	GLY 439	B	
	ASP 441	A	
	ASP 441	A	
	ASP 441	A	salt bridge
	HIS 75	A	pi-stacking
	PHE 78	B	
**malvidin**	VAL 74	B	hydrophobic contact
	PHE 78	A	
	PHE 78	A	
	PHE 78	A	
	PHE 78	B	

	TYR 407	A	
	HIS 75	A	hydrogen bonding
	HIS 75	B	
	PHE 78	A	pi-stacking
	PHE 78	B	
**cyanidin**	VAL 74	A	hydrophobic contact
	VAL 74	B	
	PHE 78	A	
	PHE 78	A	
	PHE 78	B	
	PHE 78	B	
	PHE 78	B	
	TYR 407	B	

Table S9. Mapping details of Glutathione reductase (GSH) binding sites.

**Glutathione reductase (GSH) – molecule Lansoprazole**				
**Cluster**	**affinity energy (kcal/mol)**	**nº ofposes**	**bestpose**	**site**
1	−9.0	1477	397	−13.19, −35.94, −35.25
2	−8.0	83	15	−35.91, −20.57, −32.62
3	−7.9	78	476	−35.86, −54.48, −37.44
4	−7.6	90	199	−24.13, −29.37, −15.31
5	−7.5	91	644	−24.12, −46.01, −54.72
6	−7.4	26	90	−22.27, −19.50, −32.37
7	−7.4	25	549	−22.28, −55.53, −37.59
8	−7.2	54	87	−13.54, −12.56, −20.41
9	−7.2	53	548	−13.54, −62.49, −49.55
10	−7.1	26	526	2.30, −35.62, −42.11
11	−7.1	34	65	2.29, −39.42, −27.88
12	−6.8	18	150	−17.23, −28.48, −2.83
13	−6.7	4	226	−3.54, −40.97, −20.72
14	−6.7	7	634	−3.55, −34.09, −49.25
15	−6.7	21	610	−17.19, −46.44, −67.11
16	−6.6	21	295	−27.45, −12.06, −4.23
17	−6.5	22	756	−27.46, −62.98, −65.74
18	−6.3	1	302	−37.78, −22.17, −16.30
19	−6.2	3	762	−38.09, −52.70, −53.76
20	−6.2	3	26	−46.43, −7.88, −25.14
21	−6.2	20	465	−46.45, −67.14, −44.83
22	−6.0	23	716	−12.73, −30.45, −66.64
23	−6.0	6	252	−12.73, −44.65, −3.27
24	−6.0	6	211	−27.99, −39.26, −9.44
25	−6.0	3	672	−27.99, −35.75, −60.52
26	−5.8	5	104	−32.98, −1.10, −36.37
27	−5.8	9	566	−32.99, −73.95, −33.61
28	−5.7	12	725	0.66, −53.63, −59.65
29	−5.7	2	432	−42.16, −43.27, −44.39
30	−5.6	11	243	0.05, −20.59, −13.58
31	−5.6	2	837	−38.21, −22.36, −52.37

32	−5.5	4	128	−21.79, 1.44, −12.27
33	−5.5	4	589	−21.82, −76.47, −57.71
34	−5.5	2	377	−39.50, −52.36, −16.92
35	−5.0	1	237	−10.39, −15.64, −9.05
**Glutathione reductase (GSH) – molecule Malvidin**				
**Cluster**	**affinityenergy (kcal/mol)**	**nº ofposes**	**bestpose**	**site**
1	−8.5	132	814	−10.37, −39.44, −35.69
2	−7.4	69	497	−14.55, −62.99, −50.15
3	−7.4	71	43	−14.57, −12.08, −19.80
4	−7.2	69	902	−34.12, −21.34, −32.68
5	−7.2	70	457	−34.14, −53.69, −37.30
6	−7.0	30	298	−35.64, −20.99, −12.89
7	−7.0	32	759	−35.65, −54.05, −57.08
8	−6.9	17	88	−16.84, −21.79, −36.57
9	−6.9	15	392	−16.84, −53.26, −33.41
10	−6.9	68	201	−22.92, −26.52, −13.65
11	−6.9	69	638	−22.94, −48.54, −56.30
12	−6.8	11	664	−18.27, −45.26, −64.65
13	−6.8	11	206	−18.31, −29.83, −5.39
14	−6.6	5	561	−25.29, −58.86, −37.93
15	−6.6	7	100	−25.43, −15.99, −32.08
16	−6.5	14	211	−32.81, −33.61, −17.61
17	−6.5	13	897	−32.82, −41.46, −52.22
18	−6.4	30	394	−4.57, −43.82, −22.87
19	−6.4	30	680	−4.59, −31.28, −47.13
20	−6.0	15	114	−26.85, −10.09, −4.29
21	−6.0	15	575	−26.88, −64.94, −65.70
22	−5.9	19	263	0.45, −20.69, −11.88
23	−5.9	17	724	0.43, −54.34, −58.11
24	−5.8	3	236	−11.12, −15.61, −6.53
25	−5.8	4	698	−11.16, −59.44, −63.42
26	−5.8	1	672	−24.69, −25.44, −53.17
27	−5.7	2	541	4.70, −43.73, −48.02
28	−5.7	2	80	4.70, −31.32, −21.98
29	−5.6	1	214	−27.41, −41.71, −9.30
30	−5.6	16	28	−44.88, −5.05, −19.75
31	−5.6	17	489	−44.91, −69.97, −50.21
32	−5.6	6	712	−7.54, −31.92, −63.80
33	−5.4	4	260	−7.54, −43.13, −6.17
34	−5.3	1	255	−18.37, −38.80, 4.86
35	−5.3	1	716	−18.37, −36.19, −74.84
36	−5.3	3	127	−22.61, 2.19, −11.12
37	−5.3	3	589	−22.63, −77.20, −58.86
38	−5.3	11	562	−30.14, −78.56, −37.32
39	−5.3	10	1	−30.30, 3.50, −32.77
40	−5.0	3	836	−40.48, −21.78, −52.49
41	−5.0	3	375	−40.49, −53.24, −17.44
42	−5.0	1	436	−43.12, −47.35, −40.61
43	−5.0	1	432	−43.15, −27.74, −29.31
**Glutathione reductase (GSH) – molecule Lansoprazole**				

Cluster	affinityenergy (kcal/mol)	nº ofposes	bestpose	site
1	−8.9	81	527	−9.63, −36.01, −34.37
2	−8.9	73	451	−14.95, −41.24, −36.53
3	−7.9	69	918	−34.24, −21.13, −33.39
4	−7.9	72	472	−34.26, −53.91, −36.57
5	−7.5	76	273	−14.83, −12.35, −19.59
6	−7.5	82	618	−14.86, −62.65, −50.39
7	−7.5	59	176	−27.36, −31.44, −17.51
8	−7.5	61	829	−27.63, −43.38, −52.45
9	−7.4	22	99	−24.28, −18.45, −30.99
10	−7.4	12	560	−24.30, −56.58, −38.98
11	−7.1	2	385	−16.75, −53.75, −33.71
12	−7.0	1	844	−16.80, −21.26, −36.32
13	−7.0	40	709	−17.48, −45.51, −65.05
14	−7.0	43	250	−17.48, −29.50, −4.94
15	−6.9	17	136	−35.84, −21.12, −12.43
16	−6.9	16	770	−35.87, −53.89, −57.53
17	−6.8	9	218	−4.91, −43.31, −22.73
18	−6.8	10	679	−4.94, −31.75, −47.25
19	−6.7	18	71	3.15, −34.25, −41.01
20	−6.7	17	532	3.15, −40.81, −28.98
21	−6.6	15	282	−27.27, −9.70, −4.24
22	−6.6	15	743	−27.30, −65.33, −65.74
23	−6.4	2	676	−24.18, −25.69, −53.51
24	−6.1	1	632	5.19, −45.99, −48.21
25	−6.1	13	172	−0.68, −20.27, −12.83
26	−6.1	10	633	−0.70, −54.79, −57.15
27	−6.1	3	705	−11.35, −59.05, −62.98
28	−6.1	13	26	−46.80, −9.38, −22.25
29	−6.1	14	595	−46.81, −65.68, −47.68
30	−6.0	4	255	−11.65, −44.36, −3.23
31	−6.0	5	716	−11.66, −30.70, −66.74
32	−6.0	3	237	−11.80, −15.67, −6.23
33	−6.0	2	253	−18.46, −37.93, 3.01
34	−6.0	2	714	−18.47, −37.08, −72.98
35	−6.0	7	0	−22.96, 2.43, −11.43
36	−6.0	8	461	−23.00, −77.46, −58.54
37	−6.0	8	486	−33.25, −71.34, −32.21
38	−5.9	6	105	−33.19, −3.59, −37.67
39	−5.7	2	117	−42.59, 1.84, −19.48
40	−5.7	1	580	−42.62, −76.88, −50.50
41	−5.4	3	838	−40.39, −21.83, −51.88
42	−5.4	3	377	−40.42, −53.20, −18.09
43	−5.3	1	893	−42.85, −47.29, −40.41
44	−5.3	1	432	−42.88, −27.80, −29.56

Myeloperoxidase (MPO)

Representations of the most significant structural details of this system.

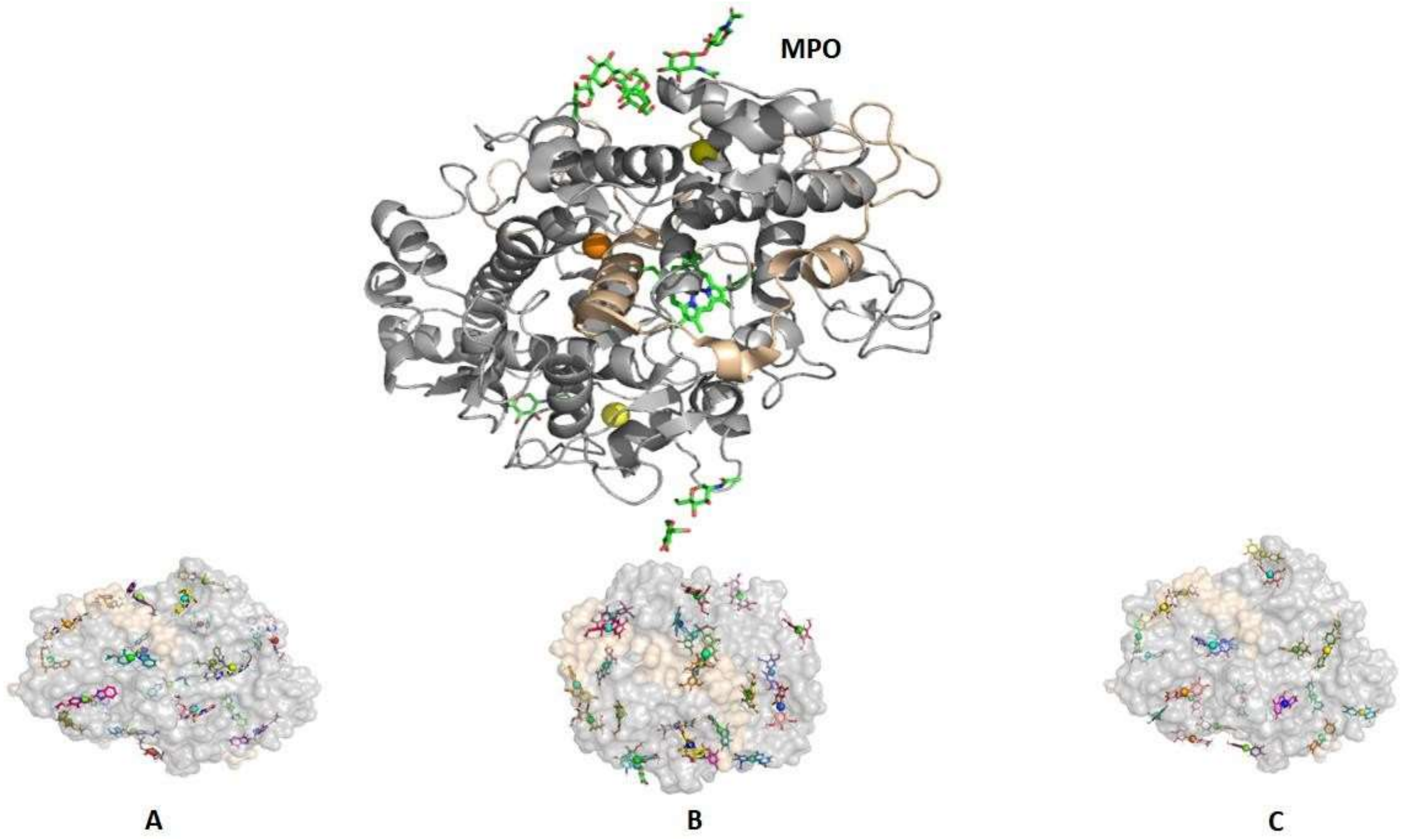

Figure S6. Structure of Myeloperoxidase (MPO) protein - PDB 7LAE, where the represen-tations: **A**, **B**, and **C**, correspond respectively to the affinity site mapping of the molecules Lansoprazole, Malvidin, and Cyanidin. Cl^- and Ca^{2+} ion represented as a yellow and or- ange colored ball respectivel; heme group; acetamido–2-deoxy-beta-D-glucopyranose; beta-D-manopyranose; alpha-D-manopyranose; and alpha-L-fucopyranose shown colored in green.

Table S10. Main molecular interactions detected in the Myeloperoxidase (MPO) protein.

Compound	residue	chain	interaction
**lansoprazole**	PRO 220	B	hydrophobic contact
	PHE 366	B	
	PHE 407	B	
	VAL 410	B	
	GLU 102	A	salt bridge
	THR 238	B	halogen bonding
**malvidin**	GLU 102	A	hydrophobic contact
	PRO 145	B	
	LEU 415	B	
**cyanidin**	PHE 99	A	hydrophobic contact
	PRO 145	B	
	LEU 415	B	

Table S11. Mapping details of Myeloperoxidase (MPO) binding sites.

Myeloperoxidase (MPO) – molecule Lansoprazole				
**Cluster**	**affinity energy (kcal/mol)**	**nº ofposes**	**bestpose**	**site**
1	−7.5	116	228	−6.07, 24.70, −23.04
2	−7.3	58	515	−2.52, −2.00, −51.39
3	−6.9	64	536	14.45, 2.66, −11.41
4	−6.9	59	487	−5.39, −10.81, −28.77
5	−6.7	66	202	17.03, 16.84, −39.87
6	−6.6	25	112	−28.14, 25.93, −24.62
7	−6.5	46	122	−22.79, 21.47, −4.57
8	−6.4	15	169	−3.81, 13.28, −49.56
9	−6.4	29	474	−4.38, −6.21, −9.40
10	−6.3	18	438	−19.28, −7.71, −8.49
11	−6.2	13	357	20.49, 19.69, −18.59
12	−6.2	8	387	7.22, 8.66, −5.25
13	−6.1	9	523	8.66, −9.71, −28.09
14	−6.1	6	312	−25.43, 12.94, −33.73
15	−6.0	20	422	−31.72, 9.30, −16.51
16	−5.9	6	88	5.06, 6.04, −31.26
17	−5.9	4	95	−9.59, 21.31, −34.61
18	−5.6	6	393	−0.47, 25.80, −5.49
19	−5.6	1	68	−9.16, 8.97, 2.81
20	−5.2	1	158	−24.38, 2.48, −36.75
21	−5.0	1	554	19.72, 2.99, −37.38
22	−4.9	1	218	9.03, 31.47, −31.93
**Myeloperoxidase (MPO) – molecule Malvidin**				
**Cluster**	**affinityenergy (kcal/mol)**	**nº ofposes**	**bestpose**	**site**
1	−7.0	104	154	−8.10, 24.08, −22.70
2	−6.7	62	288	−2.70, −9.40, −32.60
3	−6.7	53	323	−5.44, −4.67, −7.76
4	−6.5	48	342	15.38, 3.92, −11.34
5	−6.5	43	510	−9.30, −2.67, −50.13
6	−6.4	64	180	16.16, 12.39, −40.58
7	−6.3	28	11	−4.15, 12.30, −49.40
8	−6.1	4	483	−18.81, −11.47, −27.38
9	−6.0	6	165	−4.34, 22.30, −36.84
10	−6.0	28	121	−23.88, 22.00, −6.52
11	−6.0	44	464	−32.08, 8.92, −14.81
12	−5.9	16	43	−24.76, 26.49, −27.65
13	−5.9	4	315	−27.01, 13.91, −31.52
14	−5.8	15	559	10.19, −10.04, −29.74
15	−5.8	6	158	−23.93, 0.91, −36.85
16	−5.7	11	126	−2.14, 15.76, 1.01
17	−5.5	6	560	2.85, −3.96, −53.02
18	−5.5	1	127	−3.84, 29.28, −4.52
19	−5.5	2	465	−29.80, 0.41, −21.95
20	−5.4	13	218	20.34, 22.37, −17.77
21	−5.4	1	217	7.07, 31.96, −31.62

22	−5.4	18	441	−17.59, −8.36, −9.37
23	−5.1	1	325	7.97, 7.09, −5.30
24	−3.0	4	92	5.56, 5.16, −30.68
**Myeloperoxidase (MPO) – molecule Cyanidin**				
**Cluster**	**affinityenergy (kcal/mol)**	**nº ofposes**	**bestpose**	**site**
1	−7.6	106	109	−8.06, 23.94, −21.94
2	−7.4	71	494	−5.71, −10.49, −30.30
3	−7.3	65	177	16.30, 13.35, −39.94
4	−7.0	52	342	15.98, 4.83, −11.76
5	−7.0	39	43	−23.32, 23.37, −6.95
6	−6.9	50	326	−5.22, −4.96, −8.11
7	−6.9	37	14	−9.01, −2.20, −50.33
8	−6.8	18	64	−19.04, 17.83, 0.25
9	−6.7	28	165	−3.92, 12.71, −49.14
10	−6.6	15	39	−27.51, 25.64, −26.07
11	−6.4	13	92	4.62, 6.16, −31.56
12	−6.4	7	314	−23.90, 1.14, −36.86
13	−6.4	8	33	−26.49, 12.50, −31.70
14	−6.4	24	440	−31.84, 8.60, −14.39
15	−6.0	15	363	17.92, 23.86, −14.25
16	−6.0	8	552	10.92, −9.71, −29.87
17	−6.0	2	562	3.07, −4.42, −52.98
18	−6.0	2	393	−4.02, 28.89, −5.41
19	−6.0	4	70	−7.23, 9.86, 2.51
20	−5.8	6	442	−17.82, −8.10, −8.90
21	−5.8	1	465	−29.33, 0.10, −22.61
22	−5.1	1	96	−18.18, 20.64, −36.60

References

1. Ng, K.Y.; Yong, J.; Chakraborty, T.R. Estrous cycle in ob/ob and ovariectomized female mice and its relation with estrogen and leptin. *Physiol. Behav.* **2010**, *99*, 125–130, https://doi.org/10.1016/j.physbeh.2009.11.003.
2. Caligioni, C.S. Assessing reproductive status/stages in mice. *Curr. Protoc. Neurosci.* **2009**, *48*, A−4I, https://doi.org/10.1002/0471142301.nsa04is48.
3. Cora, M.C.; Kooistra, L.; Travlos, G. Vaginal Cytology of the Laboratory Rat and Mouse: Review and Criteria for the Staging of the Estrous Cycle Using Stained Vaginal Smears. *Toxicol. Pathol.* **2015**, *43*, 776–793, https://doi.org/10.1177/0192623315570339.
